# Pathobiology and first report of larval nematodes (Ascaridomorpha sp.) infecting freshwater mussels (*Villosa nebulosa*, Unionidae), including an inventory of nematode infections in freshwater and marine bivalves

**DOI:** 10.1016/j.ijppaw.2019.05.006

**Published:** 2019-06-13

**Authors:** Andrew McElwain, Micah B. Warren, Felipe B. Pereira, Steven P. Ksepka, Stephen A. Bullard

**Affiliations:** a Department of Biological Sciences, College of Liberal Arts and Sciences, State University of New York (SUNY) at Oswego, 30 Centennial Drive, Oswego, NY 13126, USA; b Aquatic Parasitology Laboratory, School of Fisheries Aquaculture and Aquatic Sciences, College of Agriculture, Auburn University, 203 Swingle Hall, Auburn, AL, 36849, USA; c Programa de Pós-Graduação em Biologia Animal, Instituto de Biociências, Universidade Federal de Mato Grossodo Sul, Av. Costa e Silva s/n°, CEP 79070-900, Campo Grande, MS, Brazil

**Keywords:** Unionidae, Histopathology, Histozoic nematodes, Ascaridomorpha

## Abstract

Little information is available on host-parasite relationships between bivalves and larval nematodes. Herein, we describe nematode larvae (likely stage 2) in the infraorder Ascaridomorpha infecting the foot, intestine, and mantle of a freshwater mussel (Alabama rainbow, *Villosa nebulosa* [Conrad, 1834]) and detail histopathological changes to infected tissues. A total of 43 live mussels from the South Fork of Terrapin Creek, Alabama, were collected between 2010 and 2014, with 14 sectioned for histopathology and 29 dissected. Of the 14 sectioned mussels, 5 appeared to be uninfected, and 7, 1, and 1 had histozoic infections observed in the foot and intestine, intestine only, and mantle edge and foot, respectively. Twenty-three of 29 (79%) of the mussels dissected were infected by live nematodes, and mean nematode abundance was 8.3 (CL = 5.23–13), with 2 mussels infected with >100 nematodes each. Thus, with a total of 32 of the 43 collected mussels observed with nematodes, overall infection prevalence was 74.4% (CL = 0.594–0.855). The 18S rDNA of this nematode was 99% similar to that of several ascaridids (species of Kathlaniidae Lane, 1914 and Quimperiidae Baylis, 1930) that mature in aquatic/semi-aquatic vertebrates; the recovered 18S phylogenetic tree indicated this nematode from *V. nebulosa* shares a recent common ancestor with *Ichthyobronema hamulatum* (Ascaridomorpha: Quimperiidae; GenBank Accession Number KY476351). Pathological changes to tissue associated with these infections comprised focal tissue damage, but a cellular response was not evident. The Alabama rainbow possibly represents an intermediate or paratenic host. Given these results, the nematode is likely not pathogenic under normal stream conditions; however, high intensity infections in the foot could inhibit pedal extension and retraction; which would have demonstrable health consequences to a freshwater mussel. Based on our review of the bivalve mollusc parasite literature, a collective biodiversity of 61 nematodes reportedly exhibit some degree of symbiosis (from commensal to parasitic) with 21 bivalves (28 nematode spp. from 17 marine bivalve spp.; 33 nematode spp. from 4 freshwater bivalve spp.); only four records exist of putatively parasitic nematodes from Unionida. The present study represents the first description of a nematode species that invades the tissues of a Unionidae species.

## Highlights

• Pathological changes comprised focal tissue damage but a cellular response to the parasites was not evident.
• Nematodes from infected tissues appear to be second stage larvae of Ascaridomorpha.
• The small subunit rDNA sequence of this nematode was 99% similar to species of Kathlaniidae and Quimperiidae.
• The nematode is likely not pathogenic under normal stream conditions.

## Graphical abstract

**Figure:**
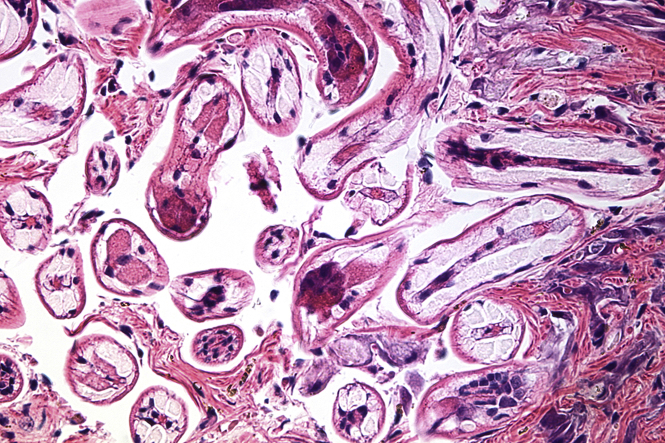

## 1 Introduction

“Freshwater mussels” are unique bivalve molluscs (Mollusca, Bivalvia, Unionida) because they are parasites of fishes during their larval period and because they use their gills for brooding glochidia, respiration, and filter feeding (Barnhart et al., 2008). North America is historically known for its high species richness of mussels comprising approximately 298 species (Margaritiferidae: 5, Unionidae: 293) (Williams et al., 2017). However, much of this fauna has declined, and as much as 71% of the mussel species across the continental U.S. may be imperiled (Williams and Neves, 1995). The dwindling of mussel populations is largely thought to stem from habitat degradation, toxic contaminants or a synergism of these problems (Hughes and Parmalee, 1999; Grabarkiewicz and Davis, 2008). Pathogens and parasitic infections could be contributing factors, but the biodiversity of metazoan parasites and other etiological agents of Unionida is understudied relative to marine bivalves (Lauckner, 1990; Grizzle and Brunner, 2009) and a direct cause-effect relationship between the presence of a given parasite in a freshwater mussel and demonstrable physiological dysfunction is typically lacking in published reports.

Based on our review of the bivalve mollusc parasite literature, conservatively 61 nematode species have been reported from 21 bivalve species (28 nematode spp. from 17 marine bivalve spp. [Table 1], 33 nematode spp. from 4 freshwater bivalve spp. [Table 2]) totaling 58 sources of literature. However, 33 articles only reported nematodes at the genus level or higher, including 6 sources in which the listed nematode species, genus, family or order was presumed or in which the authors stated that the nematode resembled a named species. Also, 11 articles did not specify a host species or listed the host at the genus level or higher (Table 1, Table 2). Also, four articles represent studies in which marine or freshwater bivalves were challenged with *Angiostrongylus cantonensis* (Cheng and Burton, 1965; Cheng, 1966; Knapp and Alicata, 1967 [Table 1]; Richards and Merritt, 1967 [Table 2]). Knapp and Alicata (1967) additionally examined *Crassostrea virginica*, *Ruditapes philippinarum*, and *Mactra thaanumi* for natural *A. cantonensis* infections, but did not observe infection. Many reports of nematodes from freshwater bivalves were observations of putatively commensal species from the shell surface or mantle cavity, and we know of at least two studies that have reported free-living nematodes from marine bivalves (Korringa, 1954; Anderson and Bourne, 1960). To the best of our knowledge, there are only four records of putatively parasitic nematodes from Unionida. Clark and Wilson (1912) and Coker et al. (1921) reported *Ascaris*-like worms infecting the alimentary canal of unspecified North American freshwater mussels from the Maumee River Basin and it is unclear whether these reports are from Unionidae and/or Margaritiferidae and from what localities. Wilson and Clark (1912) reported *Ascaris* sp. from the stomach of *Pyganodon grandis* in Indiana. More recently, Lopes et al. (2011) described *Rhaphidascaris* sp. (as *Hysterothylacium* sp.) from the pericardial cavity of *Rhipidodonta suavidicus* (as *Diplodon suavidicus* [Hyriidae]) from the Aripuana River, Brazil. Histozoic nematodes have principally been reported from marine bivalves and from a variety of tissues. Although some molluscs may serve as intermediate, definitive, or paratenic hosts for nematodes (Grewal et al., 2003; Morley, 2010), literature regarding histozoic roundworms from bivalves largely represents observations of larvae in marine bivalves and there is typically little or no information concerning gross and/or histopathology that would enable us to better understand these host-parasite relationships (e.g., Cobb, 1930; Cheng, 1975a; Sprent, 1977; Vázquez et al., 2006; Lopes et al., 2011).

**Table 1:** Free-living and parasitic nematodes (Nematoda) reported from marine bivalves (Mollusca: Bivalvia).

Bivalve			Nematode			Locality	Infection Site	Lesion	Reference
Order	Family	Species	Order	Family	Species				
Indeterminate	Indeterminate	Pearl oyster^a^	Ascaridida	Ascarididae	*Ascaris meleagrinae* (Shipley and Hornell, 1902) (as *A. meleagrina*, Kollar)^b^	Not reported	Not reported	Not reported	Örley (1882)
Ostreida	Pteriidae	*Pinctada imbricata* Röding, 1798 (as *Margaritifera vulgaris* Schum*.*)	Ascaridida	Ascarididae	*A. meleagrinae* (Shipley and Hornell, 1902)	Indian Ocean, Sri Lanka	Gonad, mantle, stomach, mouth	Encysted	Shipley and Hornell (1904)
Ostreida	Pteriidae	*P. imbricata* Röding, 1798 (as *M. vulgaris* Schum*.*)	Spirurida	Gnathostomatidae	*Echinocephalus uncinatus* (Molin, 1861) (as *Cheiracanthus uncinatus* Molin)	Indian Ocean, Sri Lanka	Adductor	Encysted; occurs in pink cysts, embedded in the nacre	Shipley and Hornell (1904)
Ostreida	Pteriidae	*P. imbricata* Röding, 1798 (as *M. vulgaris* Schum*.*)	Oxyurida	Oxyruidae	*Oxyuris* sp.^c^	Indian Ocean, Sri Lanka	Intestine	Not reported	Shipley and Hornell (1904)
Pectinida	Placunidae	*Placuna placenta* (Linnaeus, 1758)	Spirurida	Gnathostomatidae	*E. uncinatus* Molin, 1858 (as *C. uncinatus* Molin)	Indian Ocean, Sri Lanka	Not reported	Worm calcified into a pearl	Herdman and Hornell (1906)
Ostreida	Pteriidae	*P. imbricata* Röding, 1798 (as *M. vulgaris* Schum*.*)	Spirurida	Gnathostomatidae	*E. uncinatus* Molin, 1858 (as *E. gracilis*)	Indian Ocean, Sri Lanka	Adductor	Not reported	Shipley and Hornell (1906)
Pectinida	Placunidae	*P. placenta* (Linnaeus, 1758)	Spirurida	Gnathostomatidae	*E. uncinatus* Molin, 1858 (as *C. uncinatus* Molin)	Indian Ocean, Sri Lanka	Adductor	Worm encysted in adductor	Willey (1907)
Ostreida	Pinnidae	*Pinna* sp.	Spirurida	Gnathostomatidae	*E. uncinatus* Molin, 1858	Not reported	Not reported	Encysted	Baylis and Oxon (1920)
Pectinida	Pectinidae	*Pecten* sp.	Ascaridida	Anisakidae	*Paranisakis pectinis*Cobb (1930)	Atlantic Ocean, United States	Visceral mass	Not reported	Cobb (1930)
Pectinida	Pectinidae	*Argopecten irradians irradians* (Lamarck, 1819) (as *Pecten irradians*)	Ascaridida	Anisakidae	*P. pectinis*Cobb (1930)	Atlantic Ocean, United States	Visceral mass	Not reported	Gutsell (1930)
Cardiida	Veneridae	*Katelysia scalarina* (Lamarck, 1818)	Spirurida	Gnathostomatidae	*E. uncinatus* Molin, 1858	St. Vincent Gulf, Australia	Not reported	Not reported	Johnston and Mawson (1945)
Ostreida	Ostreidae	*Ostrea edulis* (Linnaeus, 1758)	Enoplida	Anticomidae	*Anticoma* acuminata (as *A. limalis*) Bastian, 1865	North Sea, Holland	Shell surface	Not reported	Korringa (1954)
Ostreida	Ostreidae	*O. edulis* (Linnaeus, 1758)	Enoplida	Leptosomatidae	*Thoracostoma figuratum* (Bastian 1865)	North Sea, Holland	Shell surface	Not reported	Korringa (1954)
Ostreida	Ostreidae	*O. edulis* (Linnaeus, 1758)	Enoplida	Oncholaimidae	*Pseudocella trichodes* (as *T. trichodes*) (Leuckart, 1849)	North Sea, Holland	Shell surface	Not reported	Korringa (1954)
Ostreida	Ostreidae	*O. edulis* (Linnaeus, 1758)	Enoplida	Enoplidae	*Enoplus communis* (Bastian, 1865)	North Sea, Holland	Shell surface	Not reported	Korringa (1954)
Ostreida	Ostreidae	*O. edulis* (Linnaeus, 1758)	Enoplida	Enoplidae	*E. brevis* Bastian (Bastian, 1865)	North Sea, Holland	Shell surface	Not reported	Korringa (1954)
Ostreida	Ostreidae	*O. edulis* (Linnaeus, 1758)	Monhysterida	Comesomatidae	*Adoncholaimus fuscus* (Bastian, 1865)	North Sea, Holland	Shell surface	Not reported	Korringa (1954)
Ostreida	Ostreidae	*O. edulis* (Linnaeus, 1758)	Enoplida	Oncholaimidae	*Oncholaimus skawensis* Ditlevsen, 1921	North Sea, Holland	Shell surface	Not reported	Korringa (1954)
Ostreida	Ostreidae	*O. edulis* (Linnaeus, 1758)	Enoplida	Oncholaimidae	*Metoncholaimus pristiurus* (Zur Strassen, 1894)	North Sea, Holland	Shell surface	Not reported	Korringa (1954)
Ostreida	Ostreidae	*O. edulis* (Linnaeus, 1758)	Enoplida	Enchelidiidae	*Eurystomatina filiforme* (de Man, 1889)	North Sea, Holland	Shell surface	Not reported	Korringa (1954)
Ostreida	Ostreidae	*O. edulis* (Linnaeus, 1758)	Enoplida	Enchelidiidae	*Symplocostoma longicolle* Bastian, 1865	North Sea, Holland	Shell surface	Not reported	Korringa (1954)
Ostreida	Ostreidae	*O. edulis* (Linnaeus, 1758)	Enoplida	Enchelidiidae	*Enchelidium marinum* Ehrenberg, 1836	North Sea, Holland	Shell surface	Not reported	Korringa (1954)
Ostreida	Ostreidae	*O. edulis* (Linnaeus, 1758)	Chromadorida	Cyatholaimidae	*Cyatholaimus demani* Filipjev, 1918	North Sea, Holland	Shell surface	Not reported	Korringa (1954)
Ostreida	Ostreidae	*O. edulis* (Linnaeus, 1758)	Chromadorida	Cyatholaimidae	*Praeacanthonchus punctatus* Micoletzky, 1924 (as *P. punctatus* [Bastian])	North Sea, Holland	Shell surface	Not reported	Korringa (1954)
Ostreida	Ostreidae	*O. edulis* (Linnaeus, 1758)	Chromadorida	Chromadoridae	*Euchromadora vulgaris (as Euchromodora vulgaris*) (Bastian, 1865)	North Sea, Holland	Shell surface	Not reported	Korringa (1954)
Ostreida	Ostreidae	*O. edulis* (Linnaeus, 1758)	Chromadorida	Chromadoridae	*Prochromadorella ditlevseni* (as *Chromadorita ditlevseni*) de Man, 1922	North Sea, Holland	Shell surface	Not reported	Korringa (1954)
Ostreida	Ostreidae	*O. edulis* (Linnaeus, 1758)	Desmodorida	Monopsthiidae	*Monoposthia costata* (as *M. costala*) (Bastian, 1865)	North Sea, Holland	Shell surface	Not reported	Korringa (1954)
Ostreida	Ostreidae	*O. edulis* (Linnaeus, 1758)	Monhysterida	Monohysteridae	*Mesotheristus setosus* (as *Theristus setosus* Buetschli) (Bütschli, 1874)	North Sea, Holland	Shell surface	Not reported	Korringa (1954)
Pholadida	Myidae	*Mya arenaria* (Linnaeus, 1758)	Oncholaimida	Oncholaimidae	*Pontonema vacillatum* Leidy 1855	Bay of Fundy, Canada	On the surface of the mantle and in the folds of the neck skin	Sometimes partially embedded in the epidermis of the neck	Anderson and Bourne (1960)
Mytilida	Mytilidae	*Mytilus edulis* (Linnaeus, 1758)	Ascaridida	Anisakidae	*Phocanema decipiens* (Krabbe, 1878)	Atlantic Ocean, Canada	Not reported	Not reported	Myers (1960)
Pholadida	Myidae	*M. arenaria* (Linnaeus, 1758) (as *M. areinaria*)	Ascaridida	Anisakidae	*P. decipiens* (Krabbe, 1878)	Atlantic Ocean, Canada	Not reported	Not reported	Myers (1960)
Ostreida	Ostreidae	*Crassostrea virginica* (Gmelin, 1791)	Indeterminate	Indeterminate	Not reported	Chesapeake Bay, United States	Leydig tissue near digestive diverticulum	Hemocytic infiltration; encapsulation	Burton (1963)
Pectinida	Pectinidae	*A. gibbus* (Linnaeus, 1758) (as *Aequipecten gibbus*)	Ascaridida	Ascarididae	*Porrocaecum pectinis* (Cobb, 1930)	Atlantic Ocean, United States	Not reported	Not reported	Hutton (1964)
Venerida	Veneridae	*Mercenaria mercenaria* (Linnaeus, 1758)^d^	Strongylida	Angiostrongylidae	*Angiostrongylus cantonensis* (Chen, 1935)	Connecticuit, United States	Not reported	Not reported	Cheng and Burton (1965)
Ostreida	Ostreidae	*C. virginica* (Gmelin, 1791)^d^	Strongylida	Angiostrongylidae	*A. cantonensis* (Chen, 1935)	Tred Avon River, United States	Not reported	Not reported	Cheng and Burton (1965)
Ostreida	Ostreidae	*C. virginica* (Gmelin, 1791)^e^	Strongylida	Angiostrongylidae	*A. cantonensis* (Chen, 1935)	Ninigret Pond, United States	Stomach, blood vessels, Leydig tissue	Leukocytes surrounding blood vessels containing larval worms	Cheng (1966)
Ostreida	Ostreidae	*C. virginica* (Gmelin, 1791)	Indeterminate	Indeterminate	Not reported	Chesapeake Bay, United States	Near digestive diverticulum	Not reported	Cheng (1967) (from material loaned by R. W. Burton)
Ostreida	Ostreidae	*C. virginica* (Gmelin, 1791)^f^	Strongylida	Angiostrongylidae	*A. cantonensis* (Chen, 1935)	Pearl Harbor, United States	Not reported	Not reported	Knapp and Alicata (1967)
Venerida	Veneridae	*Ruditapes philippinarum* (Adams and Reeve, 1850) (as *Venerupis philippinarum*)^f^	Strongylida	Angiostrongylidae	*A. cantonensis* (Chen, 1935)	Kaneohe Bay, United States	Not reported	Not reported	Knapp and Alicata (1967)
Venerida	Mactridae	*Mactra thaanumi* Dall, Bartsch and Rehder, 1938 (as *Matra* thaanumi)^g^				Ulong Island, Republic of Palau			Knapp and Alicata (1967)
Pectinida	Pectinidae	*A. gibbus* (Linnaeus, 1758)	Indeterminate	Indeterminate	Presumed to be *P. pectinis* (Cobb, 1930)	Atlantic Ocean and or Gulf of Mexico, United States	Adductor	Reported an opaque, yellowish parasite is encysted in the periphery of the adductor	Cummins, Jr., 1971
Pectinida	Pectinidae	*A. irradians* (Lamarck, 1819) (as *Aequipecten irradians*)	Ascaridida	Ascarididae	*P. pectinis* (Cobb, 1930)	Atlantic Ocean, United States	Commonly, but not always occurs in the adductor	Brownish coloration	Cheng (1973)
Ostreida	Ostreidae	*Magallana gigas* (Thünberg, 1793) (as *Crassostrea gigas*)	Spirurida	Gnathostomatidae	*E. crassostreai* Cheng, 1975	Hau Hoi Wan (Deep Bay), China	Gonoduct	Subepithelium conspicuously fibrous, preponderant hemocytes	Cheng (1975a)
Ostreida	Ostreidae	*M. gigas* (Thünberg, 1793)	Spirurida	Gnathostomatidae	*E. crassostreai* Cheng, 1975	Hau Hoi Wan (Deep Bay), China	Ovarian acini	Displacement, compression or rupturing of oocytes	Cheng (1975a)
Ostreida	Ostreidae	*M. gigas* (Thünberg, 1793)	Spirurida	Gnathostomatidae	*E. sinensis*Ko (1975)	Hau Hoi Wan (Deep Bay), China	Not reported	Not reported	Ko (1975)
Ostreida	Ostreidae	*M. gigas* (Thünberg, 1793)	Spirurida	Gnathostomatidae	*E. sinensis*Ko (1975)	Hau Hoi Wan (Deep Bay), China	Near digestive diverticula, stomach, intestine, mantle	Green spots where worms occurred	Ko et al. (1975)
Ostreida	Ostreidae	*M. gigas* (Thünberg, 1793)	Spirurida	Gnathostomatidae	*E. sinensis*Ko (1975)	Hau Hoi Wan (Deep Bay), China	Leydig tissue, gonoducts	Hemocytic infiltration, fibroplasia, erosion, metaplasia of pseudostratified columnar epithelium	Ko et al. (1975)
Cardiida	Mactridae	*S. solidissima* (Dillwyn, 1817)	Ascaridida	Anisakidae	Resembled *Paranisakiopsis pectinis* (Cobb, 1930)	Atlantic Ocean, United States	Foot and adductor	Brown worms occurred in foot and adductor	Perkins et al. (1975)
Pectinida	Pectinidae	*Chlamys* sp.	Ascaridida	Anisakidae	*S. sulcata* (Rudolphi, 1819)	Coral Sea, Australia	Adductor	Not reported	Sprent (1977)
Pectinida	Pectinidae	*A. balloti* (Bernardi, 1861)	Indeterminate	Indeterminate	Larval ascaridoid worms	Coral Sea, Australia	Adductor	Tissue near adductor was grossly caseous, yellow to orange or brown; hemocytic infiltration around encapsulated worms	Cannon (1978)
Ostreida	Pinnidae	*Pinna menkei* Reeve, 1858	Indeterminate	Indeterminate	Larval ascaridoid worms	Moreton Bay, Australia	Not reported	Not reported	Cannon (1978)
Ostreida	Spondylidae	*Spondylus sinensis* Schreibers, 1793 (as *S. ducalis*)	Indeterminate	Indeterminate	Larval ascaridoid worms	Coral Sea, Australia	Not reported	Not reported	Cannon (1978)
Cardiida	Mactridae	*S. solidissima* (Dillwyn, 1817)	Ascaridida	Anisakidae	*Sulcascaris* sp.	Atlantic Ocean, United States	Not reported	Not reported	Lichtenfels et al. (1978)
Pectinida	Pectinidae	*A. balloti* (Bernardi, 1861)	Ascaridida	Anisakidae	*S. sulcata* (Rudolphi, 1819)	Shark Bay, Australia	Adductor	Brown discoloration of adductor	Lester et al. (1980)
Pectinida	Pectinidae	*A. balloti* (Bernardi, 1861)	Spirurida	Gnathostomatidae	*Echinocephalus* sp.	Shark Bay, Australia	Not reported	Not reported	Lester et al. (1980)
Pectinida	Pectinidae	*A. gibbus* (Linnaeus, 1758)	Ascaridida	Anisakidae	*S. sulcata* (Rudolphi, 1819)^h^	Cape Canaveral, United States	Gonad, Adductor	Unspecified color change to gonad	Lichtenfels et al. (1980)
Cardiida	Mactridae	*S. solidissima* (Dillwyn, 1817)	Ascaridida	Anisakidae	*Sulcascaris* sp.^i^	Atlantic Ocean, United States	Not reported	Infection site slightly thickened; worms sometimes occurred in watery cysts	Payne et al. (1980)
Pectinida	Pectinidae	*A. balloti* (Bernardi, 1861)^j^	Ascaridida	Anisakidae	*S. sulcata* (Rudolphi, 1819)	Coral Sea, Australia	Adductor	Not reported	Berry and Cannon (1981)
Ostreida	Isognomonidae	*Isognomon ephippium* (Linnaeus, 1758) (as *Melina ephippium*)^j^	Ascaridida	Anisakidae	*S. sulcata* (Rudolphi, 1819)	Moreton Bay, Australia	Adductor, digestive gland, gonad	Not reported	Berry and Cannon (1981)
Ostreida	Pteriidae	*Pinctada* spp.^j^	Ascaridida	Anisakidae	*S. sulcata* (Rudolphi, 1819)	Moreton Bay, Australia	Adductor, digestive gland, gonad	Not reported d	Berry and Cannon (1981)
Pectinida	Pectinidae	*Argopecten ventricosus* (G. B. Sowerby II, 1842) (as *A. aequisulcatus*)	Spirurida	Gnathostomatidae	*E. pseudouncinatus* Millemann, 1951	San Juanico Bay, Mexico	Adductor	Yellow-brown spots on the adductor	McLean (1983)
Pectinida	Pectinidae	*A. gibbus* (Linnaeus, 1758)	Ascaridida	Anisakidae	*S. sulcata* (Rudolphi, 1819)	Atlantic Ocean, United States	Adductor	Not reported	Barber et al. (1987)
Pectinida	Pectinidae	*Equichlamys bifrons* (Lamarck, 1819) (as *Chlamys bifrons*)	Spirurida	Gnathostomatidae	*E. overstreeti* Deardorff and Ko (1985)	St. Vincent Gulf, Australia	Adductor	Not reported	Andrews et al. (1988)
Pectinida	Pectinidae	*Pecten albus* (Tate, 1887)	Spirurida	Gnathostomatidae	*E. overstreeti* Deardorff and Ko (1985)	St. Vincent Gulf, Australia	Adductor	Not reported	Andrews et al. (1988)
Pectinida	Pectinidae	*A. gibbus* (Linnaeus, 1758)	Ascaridida	Anisakidae	*S. sulcata* (Rudolphi, 1819)	Atlantic Ocean, United States	Adductor	Yellow-brown discoloration surrounding worms, encapsulation response included inflammatory cells, fibroblasts, connective tissue fibers	Deardorff (1989)
Cardiida	Mactridae	*S. solidissima* (Dillwyn, 1817)	Ascaridida	Anisakidae	*S. sulcata* (Rudolphi, 1819)	Atlantic Ocean, United States	Visceral mass, foot, adductor, mantle	Necrosis of adductor, extensive hemocytic infiltration remains after larvae migrate through the host	Murchelano and MacLean (1990)
Ostreida	Ostreidae	*C. virginica* (Gmelin, 1791)	Indeterminate	Indeterminate	Not reported	Atlantic Ocean, United States	Digestive gland	Hemocytes infiltrate connective tissue and surround the worms	Murchelano and MacLean (1990)
Ostreida	Ostreidae	*C. tulipa* (Lamark, 1819)	Spirurida	Gnathostomatidae	*E. sinensis*Ko (1975)	Tabounsu and Konkoure Estuaries, Republic of Guinea-Conakry	Hepatopancreas	Not reported	Machkevsky (1997)
Mytilida	Mytilidae	*Mytilus* sp.^k^	Indeterminate	Indeterminate	Not reported	Pacific Ocean, United States	Not reported	Not reported	Kim et al. (1998)
Ostreida	Ostreidae	*C. virginica* (Gmelin, 1791)	Indeterminate	Indeterminate	Not reported	Atlantic Ocean, United States	Not reported	Not reported	Kim et al. (1998)
Ostreida	Ostreidae	*Crassostrea* sp.^l^	Indeterminate	Indeterminate	Not reported	Gulf of Mexico, United States	Not reported	Not reported	Kim et al. (1998)
Ostreida	Ostreidae	*Saccostrea cuccullata* (Born, 1778)	Indeterminate	Indeterminate	Not reported	Exmouth Islands, Australia	Not reported	Not reported	Hine and Thorne (2000)
Cardiida	Cardiidae	*Cerastoderma glaucum* (Poiret, 1789)	Indeterminate	Indeterminate	Not reported	Marceddì and St. Gilla Lagoons, Sardinia	Not reported	Not reported	Figus et al. (2004)
Cardiida	Mactridae	*S. solidissima* (Dillwyn, 1817)	Indeterminate	Indeterminate	Not reported	Atlantic Ocean, United States	Digestive gland, visceral mass between body wall and underlying muscle tissue, foot, muscle tissue, gill	Frequently hemocyte infiltration was observed associated with larval nematodes	Kim and Powell (2004)
Cardiida	Cardiidae	*Cerastoderma glaucum* (Poiret, 1789)	Indeterminate	Indeterminate	Not reported	St. Gilla Lagoon, Sardinia	Mantle^m^	Not reported	Culurgioni et al. (2006)
Mytilida	Mytilidae	*Mytilus galloprovincialis* (Lamarck, 1819)	Indeterminate	Indeterminate	Not reported	St. Gilla Lagoon, Sardinia	Mantle^m^	Not reported	Culurgioni et al. (2006)
Cardiida	Veneridae	*Tapes decussatus* (Linnaeus, 1758)	Indeterminate	Indeterminate	Not reported	St. Gilla Lagoon, Sardinia	Mantle^m^	Not reported	Culurgioni et al. (2006)
Cardiida	Cardiidae	*Cerastoderma glaucum* (Poiret, 1789)	Indeterminate	Indeterminate	Not reported	St. Gilla Lagoon, Sardinia	Not reported	Not reported	Figus et al. (2006)
Ostreida	Ostreidae	*Crassostrea* spp*.*^l^	Indeterminate	Indeterminate	Not reported	Atlantic Ocean, United States and Puerto Rico	Not reported	Not reported	Kim and Powell (2006)
Ostreida	Ostreidae	*C. virginica* (Gmelin, 1791)	Indeterminate	Indeterminate	Not reported	Gulf of Mexico, United States	Not reported	Not reported	Kim and Powell (2006)
Mytilida	Mytilidae	*Mytilus edulis* (Linnaeus, 1758)	Indeterminate	Indeterminate	Not reported	Wadden Sea, Germany	Not reported	Not reported	Thieltges et al. (2006)
Cardiida	Psammobiidae	*Tagelus plebeius* (Lightfoot, 1787)	Spirurida	Spiruidae	Spirurine larvae	Mar Chiquita Coastal Lagoon, Mouth of Quequeén River, Brazil	Muscular wall of visceral mass, labial palps, siphon retractor muscles, radial muscles of the mantle border, mantle	Larvae may be free or encapsulated; brown spots occur where larvae are encapsulated; capsule formed by hemocytes and in some cases, bundles of muscle fibers	Vázquez et al. (2006)
Mytilida	Mytilidae	*Modiolus barbatus* (Linnaeus, 1758)	Indeterminate	Indeterminate	Not reported	Mali Ston Bay, Croatia	Mantle cavity	Not reported	Mladineo (2008)
Arcida	Arcidae	*Andara natalensis* (Krauss, 1848) (as *Scapharca natalensis*)	Spirurida	Gnathostomatidae	*Echinocephalus* sp.	Arabian Sea, Pakistan	Visceral organs, especially attached to the foot, wall of alimentary canal, and gonoduct	Not reported	Moazzam and Moazzam (2014)

a Worms may have been specimens at the British Museum.

b Shipley and Hornell (1904) suggested Kollar was a misspelling of Kelaart.

c Two specimens of *Oxyuris* sp. were observed, but specimens were lost before a species description could be made.

d *Crassostrea virginica* and *Mercenaria mercenaria* were experimentally infected with first stage larvae of *Angiostrongylus cantonensis*, second and or third stage larvae were later recovered from each host species.

e *Crassostrea virginica* was experimentally infected with first stage larvae of *Angiostrongylus cantonensis*.

f *Venerupis philippinarum*, and *Crassostrea virginica* were experimentally infected with first stage larvae of *Angiostrongylus cantonensis*, but infections largely failed and the few remaining nematodes in tissue were first stage larvae. *V. philippinarum*, and *C. virginica* were also examined for natural infections with *A. cantonensis* and were not infected.

g Examined for natural infections with *Angiostrongylus cantonensis*. No natural infections observed.

h Larval nematodes were indistinguishable from *Sulcascaris sulcata* larvae.

i Presumed to be *Sulcascaris* sp. based on Lichtenfels et al. (1978).

j *Melina ephippium* and *Pinctada* spp. were experimentally infected with *Sulcascaris sulcata*. Whereas *Amusium balloti* was naturally infected with *S. sulcata*.

k *Mytilus californianus* and *M. edulis* were sampled were sampled, but the authors did not disclose whether nematodes occurred in one or both *Mytilus* spp.

l *Crassostrea virginica* and *C. rhizophorae* were sampled, but the authors did not disclose whether nematodes occurred in one or both *Crassostrea* spp.

m Did not specify if worms were attached to the surface of the mantle or if they were embedded in tissue.

**Table 2:** Free-living and parasitic nematodes (Nematoda) reported from freshwater bivalves (Mollusca, Bivalvia).

Bivalve			Nematode					
Order	Family	Species	Order	Family	Species	Locality	Site	Reference
Indeterminate	Indeterminate	Not reported	Indeterminate	Indeterminate	*Ascaris*-like worms	Maumee River Basin, United States	Alimentary canal	Clark and Wilson (1912)
Unionida	Unionidae	*Pyganodon grandis* (Say, 1829) (as *Anodonta grandis*)	Ascaridida	Ascaridae	*Ascaris* sp.	Pretty Lake, United States	Stomach (reported as stomach contents)	Wilson and Clark (1912)
Indeterminate	Not reported	Not reported	Indeterminatre	Indeterminate	*Ascaris*-like worms	Not specified	Intestine	Coker et al. (1921)
Sphaeriida	Sphaeriidae	*Pisidium casertanum* (Poli, 1791) (as *Pisidium abditum* (Haldeman, 1841))^a^	Strongylida	Angiostrongylidae	*Angiostrongylus cantonensis* (Chen, 1935)	Not reported	Not reported	Richards and Merritt (1967)
Cariida	Dreissenidae	*Dreissena polymorpha* (Pallas, 1771)	Enoplida	Enolopidae	Free-living enolopids	Lake St. Clair, United States	Mantle cavity	Toews et al. (1993)
Cariida	Dreissenidae	*Dreissena polymorpha* (Pallas, 1771)	Enoplida	Enolopidae	Free-living enolopids	Lake Erie, Canada	Mantle cavity	Toews et al. (1993)
Cariida	Dreissenidae	*D. polymorpha* (Pallas, 1771)	Mononchida	Mononchidae	*Mononchus* sp.	St. Lawrence River, United States and Canada	Not reported	Conn et al. (1994)
Cariida	Dreissenidae	*D. polymorpha* (Pallas, 1771)	Indeterminate	Indeterminate	Three unidentified species	St. Lawrence River, United States and Canada	Not reported	Conn et al. (1994)
Cariida	Dreissenidae	*D. polymorpha* (Pallas, 1771)	Dorylaimida	Dorylaimidae	*Dorylaimus* (as *Dorylamus*) *stagnalis*	Volga River, Russia	Not reported	Kuperman et al. (1994)
Cariida	Unionidae	*Amblema plicata* (Say, 1817)	Dorylaimida	Dorylaimidae	*Dorylaimus* sp.	Kentucky Lake, United States	Shell	Benz and Curran (1997)
Cariida	Unionidae	*Fusconaia ebena* (Lea, 1831)	Dorylaimida	Dorylaimidae	*Dorylaimus* sp.	Kentucky Lake, United States	Shell	Benz and Curran (1997)
Cariida	Unionidae	*F. flava* (Rafinesque, 1820)	Dorylaimida	Dorylaimidae	*Dorylaimus* sp.	Kentucky Lake, United States	Shell	Benz and Curran (1997)
Unionida	Unionidae	*Obliquaria (*as *Quadrula) metanerva* Rafinesque, 1820 as *Quadrula metanevra*	Dorylaimida	Dorylaimidae	*Dorylaimus* sp.	Kentucky Lake, United States	Shell	Benz and Curran (1997)
Unionida	Unionidae	*Q. quadrulla* (Rafinesque, 1820)	Dorylaimida	Dorylaimidae	*Dorylaimus* sp.	Kentucky Lake, United States	Shell	Benz and Curran (1997)
Cariida	Dreissenidae	*D. polymorpha* (Pallas, 1771)	Indeterminate	Indeterminate	Not reported	Lake Erie, United States	Not reported	Kim et al. (1998)
Cariida	Dreissenidae	*D. polymorpha* (Pallas, 1771)	Indeterminate	Indeterminate	One or more unidentified free living species	Svisloch River, Belarus	Mantle cavity	Karatayev et al. (2000)
Cariida	Dreissenidae	*D. polymorpha* (Pallas, 1771)	Indeterminate	Indeterminate	One or more unidentified free living species	Dnieoper-Bug Canal, Belarus	Mantle cavity	Karatayev et al. (2000)
Cariida	Dreissenidae	*D. polymorpha* (Pallas, 1771)	Indeterminate	Indeterminate	One or more unidentified free living species	Lake Lepelskoe, Belarus	Mantle cavity	Karatayev et al. (2000)
Cariida	Dreissenidae	*D. polymorpha* (Pallas, 1771)	Indeterminate	Indeterminate	One or more unidentified free living species	Lake Lukomskoe, Belarus	Mantle cavity	Karatayev et al. (2000)
Cariida	Dreissenidae	*D. polymorpha* (Pallas, 1771)	Indeterminate	Indeterminate	One or more unidentified free living species	Lake Drivyaty, Belarus	Mantle cavity	Karatayev et al. (2000)
Cariida	Dreissenidae	*D. polymorpha* (Pallas, 1771)	Indeterminate	Indeterminate	One or more unidentified free living species	Lake Severnyi Voloso, Belarus	Mantle cavity	Karatayev et al. (2000)
Cariida	Dreissenidae	*D. polymorpha* (Pallas, 1771)	Indeterminate	Indeterminate	One or more unidentified free living species	Lake Strusto, Belarus	Mantle cavity	Karatayev et al. (2000)
Cariida	Dreissenidae	*D. polymorpha* (Pallas, 1771)	Indeterminate	Indeterminate	One or more unidentified free living species	Lake Voiso, Belarus	Mantle cavity	Karatayev et al. (2000)
Cariida	Dreissenidae	*D. polymorpha* (Pallas, 1771)	Indeterminate	Indeterminate	One or more unidentified free living species	Lake Bolduk, Belarus	Mantle cavity	Karatayev et al. (2000)
Cariida	Dreissenidae	*D. polymorpha* (Pallas, 1771)	Indeterminate	Indeterminate	One or more unidentified free living species	Lake Dolzha, Belarus	Mantle cavity	Karatayev et al. (2000)
Cariida	Dreissenidae	*D. polymorpha* (Pallas, 1771)	Indeterminate	Indeterminate	One or more unidentified free living species	Lake Lotviny, Belarus	Mantle cavity	Karatayev et al. (2000)
Cariida	Dreissenidae	*D. polymorpha* (Pallas, 1771)	Indeterminate	Indeterminate	One or more unidentified free living species	Lake Myadel, Belarus	Mantle cavity	Karatayev et al. (2000)
Cariida	Dreissenidae	*D. polymorpha* (Pallas, 1771)	Indeterminate	Indeterminate	One or more unidentified free living species	Lake Malye Shvakshty, Belarus	Mantle cavity	Karatayev et al. (2000)
Cariida	Dreissenidae	*D. polymorpha* (Pallas, 1771)	Indeterminate	Indeterminate	One or more unidentified free living species	Lake Bolshiye Shvakshty, Belarus	Mantle cavity	Karatayev et al. (2000)
Cariida	Dreissenidae	*D. polymorpha* (Pallas, 1771)	Indeterminate	Indeterminate	One or more unidentified free living species	Lake Spory, Belarus	Mantle cavity	Karatayev et al. (2000)
Cariida	Dreissenidae	*D. polymorpha* (Pallas, 1771)	Indeterminate	Indeterminate	One or more unidentified free living species	Lake Svir, Belarus	Mantle cavity	Karatayev et al. (2000)
Cariida	Dreissenidae	*D. polymorpha* (Pallas, 1771)	Indeterminate	Indeterminate	One or more unidentified free living species	Lake Vlochin, Belarus	Mantle cavity	Karatayev et al. (2000)
Cariida	Dreissenidae	*D. polymorpha* (Pallas, 1771)	Chromadorida	Chromadoridae	*Chromadorina bioculata*	Svisloch River, Belarus	Not reported	Karatayev et al. (2003)
Cariida	Dreissenidae	*D. polymorpha* (Pallas, 1771)	Monhysterida	Monhysteridae	*Eumonhystera vulgaris*	Svisloch River, Belarus	Not reported	Karatayev et al. (2003)
Cariida	Dreissenidae	*D. polymorpha* (Pallas, 1771)	Monhysterida	Monhysteridae	*Tridentulus floreanae*	Svisloch River, Belarus	Not reported	Karatayev et al. (2003)
Cariida	Dreissenidae	*D. polymorpha* (Pallas, 1771)	Enoplida	Tobrilidae	*Tobrilus tenuicadatus*	Svisloch River, Belarus	Not reported	Karatayev et al. (2003)
Cariida	Dreissenidae	*D. polymorpha* (Pallas, 1771)	Monhysterida	Monhysteridae	*Monhystrella* sp.	Svisloch River, Belarus	Not reported	Karatayev et al. (2003)
Cariida	Dreissenidae	*D. polymorpha* (Pallas, 1771)	Plectida	Plectidae	*Plectus cirratus* Bastian, 1865	Lakes Myastro and Batorino, Belarus^b^	Mantle cavity	Mastitsky and Gagarin, 2004
Cariida	Dreissenidae	*D. polymorpha* (Pallas, 1771)	Plectida	Plectidae	*P. palustris* de Man, 1880	Lake Myastro, Belarus	Mantle cavity	Mastitsky and Gagarin, 2004
Cariida	Dreissenidae	*D. polymorpha* (Pallas, 1771)	Chromadorida	Chromadoridae	*Chromadorita leuckarti* (de Man, 1876)	Lakes Myastro, Naroch, and Batorino, Belarus	Mantle cavity	Mastitsky and Gagarin, 2004
Cariida	Dreissenidae	*D. polymorpha* (Pallas, 1771)	Chromadorida	Chromadoridae	*Chromadorina bioculata* (Schultze in Carus, 1857)	Lakes Myastro, Naroch, and Batorino, Belarus	Mantle cavity	Mastitsky and Gagarin, 2004
Cariida	Dreissenidae	*D. polymorpha* (Pallas, 1771)	Chromadorida	Chromadoridae	*Punctodora ratzeburgensis* (Linstow, 1876)	Lakes Myastro and Batorino, Belarus	Mantle cavity	Mastitsky and Gagarin, 2004
Cariida	Dreissenidae	*D. polymorpha* (Pallas, 1771)	Dorylaimida	Actinolaimidae	*Neoactinolaimus dzjubani* Gagarin, 1979	Lakes Myastro, Naroch, and Batorino, Belarus	Mantle cavity	Mastitsky and Gagarin, 2004
Cariida	Dreissenidae	*D. polymorpha* (Pallas, 1771)	Dorylaimida	Dorylaimidae	*Crocodorylaimus flavomaculatus* (Linstow, 1876)	Lakes Myastro, Naroch, and Batorino, Belarus	Mantle cavity	Mastitsky and Gagarin, 2004
Cariida	Dreissenidae	*D. polymorpha* (Pallas, 1771)	Dorylaimida	Dorylaimidae	*Dorylaimus stagnalis* Dujardin, 1848	Lakes Myastro and Naroch, Belarus	Mantle cavity	Mastitsky and Gagarin, 2004
Cariida	Dreissenidae	*D. polymorpha* (Pallas, 1771)	Enoplida	Rhabdolaimidae	*Rhabdolaimus terrestris* de Man, 1880	Lakes Myastro and Naroch, Belarus	Mantle cavity	Mastitsky and Gagarin, 2004
Cariida	Dreissenidae	*D. polymorpha* (Pallas, 1771)	Triplpnchida	Trypylidae	*Tripyla glomerans* Bastian, 1865	Lakes Myastro and Naroch, Belarus	Mantle cavity	Mastitsky and Gagarin, 2004
Cariida	Dreissenidae	*D. polymorpha* (Pallas, 1771)	Enoplida	Trobrilidae	*Brevitobrilus stenfanskii* (Micoletzky, 1925)	Lakes Myastro and Batorino, Belarus	Mantle cavity	Mastitsky and Gagarin, 2004
Cariida	Dreissenidae	*D. polymorpha* (Pallas, 1771)	Enoplida	Trobrilidae	*Epitobrilus medius* (Schneider, 1916)	Lake Batorino, Belarus	Mantle cavity	Mastitsky and Gagarin, 2004
Cariida	Dreissenidae	*D. polymorpha* (Pallas, 1771)	Enoplida	Trobrilidae	*Semitobrilus gagarini* (Ebsary, 1982)	Lake Naroch, Belarus	Mantle cavity	Mastitsky and Gagarin, 2004
Cariida	Dreissenidae	*D. polymorpha* (Pallas, 1771)	Enoplida	Trobrilidae	*Tobrilus helveticus* (Hofmaenner, 1914)	Lake Myastro, Belarus	Mantle cavity	Mastitsky and Gagarin, 2004
Cariida	Dreissenidae	*D. polymorpha* (Pallas, 1771)	Monhysterida	Monhysteridae	*Eumonhystera pseudobulbosa* (Daday, 1896)	Lake Myastro, Belarus	Mantle cavity	Mastitsky and Gagarin, 2004
Cariida	Dreissenidae	*D. polymorpha* (Pallas, 1771)	Monhysterida	Monhysteridae	*E. vulgaris* (de Man, 1880)	Lake Myastro, Belarus	Mantle cavity	Mastitsky and Gagarin, 2004
Cariida	Dreissenidae	*D. polymorpha* (Pallas, 1771)	Monhysterida	Monhysteridae	*Monhystera uncispiculatum* Gagarin, 1979	Lakes Myastro and Batorino, Belarus	Mantle cavity	Mastitsky and Gagarin, 2004
Cariida	Dreissenidae	*D. polymorpha* (Pallas, 1771)	Monhysterida	Monhysteridae	*M. paludicola* de Man, 1881	Lake Myastro, Belarus	Mantle cavity	Mastitsky and Gagarin, 2004
Cariida	Dreissenidae	*D. polymorpha* (Pallas, 1771)	Monhysterida	Monhysteridae	*Monhystera lemani* Juget, 1969	Lake Myastro, Belarus	Mantle cavity	Mastitsky and Gagarin, 2004
Cariida	Dreissenidae	*D. polymorpha* (Pallas, 1771)	Monhysterida	Monhysteridae	*Tridentulus floreana*e (Eyualem, Coomans, 1995)	Lake Naroch, Belarus	Mantle cavity	Mastitsky and Gagarin, 2004
Cariida	Dreissenidae	*Dreissena* spp.	Indeterminate	Indeterminate	Not reported	Not specified; Sampled the Great Lakes and Hudson River, United States	Not reported^c^	Kim and Powell (2006)
Cariida	Dreissenidae	*D. polymorpha* (Pallas, 1771)	Dorylaimida	Dorylaimidae	*Laimydorus* sp.	Lake Erken, Sweden	Mantle cavity	Mastitsky et al. (2008)
Cariida	Dreissenidae	*D. polymorpha* (Pallas, 1771)	Chromadorida	Chromadoridae	*Chromadorina bioculata*	Lake Erken, Sweden	Mantle cavity	Mastitsky et al. (2008)
Cariida	Dreissenidae	*D. polymorpha* (Pallas, 1771)	Chromadorida	Chromadoridae	*C. leukarti*	Lake Erken, Sweden	Mantle cavity	Mastitsky et al. (2008)
Cariida	Dreissenidae	*D. polymorpha* (Pallas, 1771)	Chromadorida	Chromadoridae	*Punctodora ratzeburgensis*	Lake Erken, Sweden	Mantle cavity	Mastitsky et al. (2008)
Unionida	Hyriidae	*Rhipidodonta suavidicus* Lea, 1856 (as *Diplodon suavidicus*)	Ascaridida	Anisakidae	*Rhaphidascaris* sp. (as *Hysterothylacium* sp.)	Aripuana River, Brazil	Pericardial cavity	Lopes et al. (2011)
Cariida	Dreissenidae	*D. bugensis* (Andrusov, 1897)	Chromadorida	Achromadoridae	*Achromadora* sp.	Copper Basin Reservoir, and Lake Skinner, United States	Mantle cavity	Reid et al. (2012)
Cariida	Dreissenidae	*D. bugensis* (Andrusov, 1897)	Chromadorida	Chromadoridae	*C. bioculata*	Copper Basin Reservoir, and Lake Skinner, United States	Mantle cavity	Reid et al. (2012)
Cariida	Dreissenidae	*D. bugensis* (Andrusov, 1897)	Chromadorida	Chromadoridae	*Dichromadora* sp.	Copper Basin Reservoir, United States	Mantle cavity	Reid et al. (2012)
Cariida	Dreissenidae	*D. bugensis* (Andrusov, 1897)	Rhabditina	Diplogasteridae	*Diplogaster* sp.	Copper Basin Reservoir, United States	Mantle cavity	Reid et al. (2012)
Cariida	Dreissenidae	*D. bugensis* (Andrusov, 1897)	Enoplida	Ironidae	*Ironus* sp.	Copper Basin Reservoir, United States	Mantle cavity	Reid et al. (2012)
Cariida	Dreissenidae	*D. bugensis* (Andrusov, 1897)	Dorylaimida	Dorylaimidae	*Laimydorus* sp. A	Lake Skinner, United States	Mantle cavity	Reid et al. (2012)
Cariida	Dreissenidae	*D. bugensis* (Andrusov, 1897)	Dorylaimida	Dorylaimidae	*Laimydorus* sp. B	Copper Basin Reservoir, United States	Mantle cavity	Reid et al. (2012)
Cariida	Dreissenidae	*D. bugensis* (Andrusov, 1897)	Monhysterida	Monhysteridae	*Monhystrella* sp.	Lake Skinner, United States	Mantle cavity	Reid et al. (2012)
Cariida	Dreissenidae	*D. bugensis* (Andrusov, 1897)	Plectida	Plectidae	*Plectus geophilus*	Copper Basin Reservoir, United States	Mantle cavity	Reid et al. (2012)
Cariida	Dreissenidae	*D. bugensis* (Andrusov, 1897)	Enoplida	Rhabdolaimidae	*Rhabdolaimus* sp.	Copper Basin Reservoir, United States	Mantle cavity	Reid et al. (2012)
Cariida	Dreissenidae	*D. bugensis* (Andrusov, 1897)	Aphelenchida	Aphelenchoididae	*Seinura* sp.	Copper Basin Reservoir, United States	Mantle cavity	Reid et al. (2012)

a Host was experimentally infected with *Angiostrongylus cantonensis*.

b Lakes Myastro, Naroch, and Batorino are connected.

c The authors observed nematodes in histological sections, but did not report the infection site.

Much of the literature about parasites in freshwater mussels concerns members of Unionidae, consisting of biodiversity surveys using a gross inspection of tissues or studies having a taxonomic focus (Grizzle and Brunner, 2009). Few investigations have used histology to characterize host-parasite relationships at the cellular level (Antipa and Small, 1971; Huehner and Etges, 1981; Müller et al., 2015; McElwain et al., 2016). These gaps in our knowledge represent a barrier to our understanding of mussel health. Given the above, the lack of histopathological studies on parasites of mussels in freshwater habitats is a bottleneck to our understanding of species declines.

While describing the tissues of *Villosa nebulosa* towards producing the first unionid histological atlas (McElwain and Bullard, 2014), small nematodes were observed in the foot and other tissues. Herein we describe histopathological changes to the foot, and intestine of *V. nebulosa* from Alabama – an investigation that represents the first description of a nematode species that invades the tissues of a Unionidae species.

## 2 Materials and methods

### 2.1 Mussel collections

Mussels were collected from the South Fork of Terrapin Creek near the Cleburne County Road 55 crossing (N33°51′36.56” W85°31′28.15”) in May 2010 (n = 11), August 2011 (n = 5), June 2012 (n = 2), May 2013 (n = 16), June 2014 (n = 9). All mussels observed were live and were collected by hand while snorkeling, transported to Auburn University in an aerated cooler filled with stream water from the collection site.

### 2.2 Histological processing

The mussels sampled for histology consisted of 14 individuals. Regarding histological methodology, the valves of each mussel were propped open with wooden dowels to facilitate proper fixation. Mussels were immersed in 10% neutral buffered formalin for 48 h, rinsed in tap water to remove buffer salts, and dehydrated in a graded ethanol series. Formalin fixed mussels were removed from their shells by excising soft tissues from the nacre using a scalpel and divided into pieces by cutting through the visceral mass with a grossing knife. Each sample was processed for routine paraffin embedding using the Tissue-Tek^®^ Mega-Cassette^®^ System and a Tissue-Tek VIP E300 (Sakura^®^ Finetek, Inc., Tokyo, Japan) automated tissue processor. Following tissue processing, pieces of visceral mass were embedded using a Tissue-Tek Thermal Console 4585/7 (Sakura Finetechnical Co., LTD, Tokyo, Japan).

Before sectioning, paraffin blocks were immersed for 1 min in an ice-water mixture immediately before sectioning. Paraffin blocks were sectioned at 4 μm thickness using a Reichert-Jung Biocut 2030 (Wetzlar, Germany), immediately thereafter moved to a Boekel Scientific 145701 lighted tissue floatation bath water (Feasterville, Pennsylvania) at 43 °C and pre-mixed with histology adhesive and lifted with forceps. Slides with paraffin sections were placed into a stainless steel 50 slide staining rack and heated to 63 °C for 45 min to remove excess paraffin and stained in a Sakura Finetek automated slide stainer with fume hood (Tiyoda MFG, USA, Torrance, California) using Harris's hematoxylin and eosin as per Luna (1968). Stained slides were photographed using a digital single lens reflex camera mounted on a Leica DM 2500 compound microscope (Wetzlar, Germany).

### 2.3 Mussel dissection

A total of 29 individuals were necropsied to obtain nematodes for pathology and for taxonomic diagnosis based on morphological and phylogenetic molecular analyses. Approximately 5 mm^3^ pieces of tissue were excised from the foot of live mussels using straight dissecting scissors. Each piece of tissue was placed into a Petri dish filled with deionized water and subdivided into smaller pieces using a scalpel. Small samples of pedal tissue were then wet mounted and gently compressed between two 10 × 8 x ¼ inch plates of glass. Compressed tissues were carefully inspected for roundworms with a Meiji Techno RZDT stereomicroscope (Meiji Techno Co., Ltd., San Jose, California) at a high magnification under bright field and dark field illumination. Infected pieces were removed from the plates and reserved in a small dish filled with deionized water while uninfected pieces were discarded. Each infected piece of tissue was gently teased apart using fine-tipped forceps. Fibrous tissue was carefully removed from the vicinity of each worm, using 0.20 mm diameter BioQuip Minuten pins, each mounted in a BioQuip pin vise. Individual worms were transferred to a separate dish of deionized water and allowed to crawl freely to remove any attached debris.

### 2.4 Nematode processing and taxonomic identification

For a morphological diagnosis, worms were fixed in a small dish containing glacial acetic acid until they became straightened, then transferred to a vial containing 70% ethanol. Nematodes intended as whole-mounts were photographed with the aid of a stereo-dissecting microscope and fiber optic light source, rinsed with distilled water, immersed in 95% glacial acetic acid for 5–10 min, fixed and cleared in 5 parts glycerin plus 95 parts 70% ethanol (EtOH) (“70 + 5”), mounted on glass slides using glycerin jelly, and studied with a Leica DM 2500 microscope with differential interference contrast (DIC) optical components.

For gene sequencing, worms were fixed in 2.0 ml cryo-storage vials containing 95% ethanol or RNA*later™* and stored at −20 °C. Using the pooled (4) EtOH-preserved and microscopically-identified nematodes from Alabama rainbow, total genomic DNA (gDNA) was extracted using DNeasyTM Blood and Tissue Kit (Qiagen, Valencia, California, USA) as per the manufacturer's protocol with one exception: the proteinase-K incubation period was extended overnight and the final elution step used 100 μl of elution buffer to increase the final DNA concentration. Inhibitors were removed from extracted DNA using OneStepTM PCR Inhibitor Removal Kit (Zymo Research, Irvine, California, USA). Amplification and sequencing of the small subunit ribosomal DNA (18S) used the set of primers described in Floyd et al. (2005). PCR amplifications were performed using a total volume of 25 μl with 2 μl of DNA template, 0.2 μM of each primer along with 1 × buffer, 3 mM MgCl_2_, 0.2 mM dNTP mixture, and 0.15 μl Taq polymerase (5 U/μl) (Promega, Madison, Wisconsin, USA). The thermocycling profile comprised 5 min at 94 °C for denaturation, 35 repeating cycles at 94 °C for 30 s for denaturation, 54 °C for 30 s for annealing, and 72 °C for 1 min for extension followed by a final 10 min at 72 °C for extension. All PCR reactions were carried out in a MJ Research PTC-200 (BioRad, Hercules, California, USA). PCR products (10 μl) were verified on a 1% agarose gel and stained with ethidium bromide. PCR products were purified by microcentrifugation with the QIAquick PCR Purification Kit (Qiagen, Valencia, California, USA) according to manufacturer's protocols except that the last elution step was performed with autoclaved nanopure H_2_O rather than with the provided buffer. DNA sequencing was performed by ACGT, Incorporated (Wheeling, Illinois, USA). Reactions were sequenced using BigDye terminator version 3.1, cleaned with magnetic beads (CleanSeq dye terminator removal kit), and analyzed using an ABI 3730 XL or 3730 Genetic Analyzer. Sequence assembly and analysis of the chromatogram was performed with Geneious version 11.0.5 (http://www.geneious.com). All nucleotide sequence data were deposited in GenBank.

A preliminary NCBI BLAST (https://blast.ncbi.nlm.nih.gov) search showed high genetic similarity (>97%) between the new sequence and those of Cosmocercoidea and Seuratoidea; therefore representatives of these superfamilies were used in the phylogenetic analysis. The dataset for phylogeny consisted of 23 taxa belonging to Atractidae, Cosmocercidae and Kathlaniidae (Cosmocercoidea), and Cucullanidae, Quimperiidae and Seuratidae (Seuratoidea) plus *Zeldia punctata* (Thorne, 1925) (Cephaloboidea: Cephalobidae) as outgroup, chosen according to previous studies (Choudhury and Nadler, 2016; Pereira and Luque, 2017; Sokolov and Malysheva, 2017). Sequences with less than 800bp and without genetic overlapping were excluded. Except for Cucullanidae, which is considered monophyletic (Choudhury and Nadler, 2016), all available taxa assigned to Cosmocercoidea and Seuratoidea were included in the analysis. Sequences were aligned using T-Coffee (Notredame et al., 2000), then evaluated by the transitive consistency score, to verify the reliability of aligned positions and, based on score values, ambiguous aligned positions were trimmed (Chang et al., 2014). The phylogenetic tree was generated in MRBAYES (Huelsenbeck and Ronquist, 2001), using Bayesian inference and nodal supports estimated by Bayesian posterior probability after running the Markov chain Monte Carlo (2 runs 4 chains) for 4 × 10^6^ generations, with sampling frequency every 4 × 10^3^ generations and discarding the initial ¼ of sampled trees (1 × 10^6^) as burn-in. The model of evolution (TIM3+I + G) and its fixed parameters for phylogenetic reconstruction were chosen and estimated under the Akaike informative criterion with jModelTest 2 (Guindon and Gascuel, 2003; Darriba et al., 2012).

Each mussel was identified to species by the anatomical diagnostic features provided by Burch (1973), and Williams et al. (2008). Nomenclature and higher-level systematics of bivalves follows Asghari et al. (2017), Bieler et al. (2010), Cunha et al. (2011), Huber (2010), Mikkelsen et al. (2006), Mackie (2007), Puslednik and Serb (2008), Saavedra and Peña (2004), Salvi et al. (2014), and Williams et al. (2017). Nomenclature and higher-level systematics of nematodes follows Abebe et al. (2006), Anderson et al. (2009), Bongers (1983), Boufahja et al. (2014), Deardorff et al. (1981), Eisendle (2008), Gagarin and Gusakov (2016), Gagarin and Naumova (2017), Greenslade (1989), Holovachov (2014), Miljutina and Miljutin (2015), Moravec (1998), Semprucci (2013), Sharma et al. (2006), Tahseen and Mustaqim (2011), Traunspurger (2000), Warwick (1971), and Zograf et al. (2008).

### 2.5 Statistical analyses

Prevalence and mean abundance values defined according to Bush et al. (1997), were calculated in the Quantitative Parasitology Program (QPweb Version 1.0.14, Reiczigel et al., 2019). The 95% confidence limits (CL) of prevalence were calculated using the Sterne's exact method, and those for mean abundance were calculated using bootstrapping with 2000 replications.

## 3 Results and discussion

### 3.1 Prevalence, abundance, and site of infections

From the total sample of mussels, the prevalence of nematodes was 74.4% (CL = 0.594–0.855, n = 43). Of the 14 individuals examined with histology, 9 were infected. Of these 9 individuals, 7 mussels displayed nematodes in the foot and intestine, one individual presented nematodes only in the intestine, and one individual had nematodes in the mantle edge and foot. From the sample of 29 individuals necropsied, 23 were infected. Based on dissections, intensity ranged from 0 to 39. Mean abundance was 8.3 (CL = 5.23–13, n = 26). However, two of the 29 individuals were infected with >100 nematodes, and one additional infected individual was excluded from this analysis because its tissues were placed directly into a 2.0 ml cryo-storage vial containing RNA*later™*. Nematodes were more abundant in the foot than in other tissues, and therefore we focused our attention on extricating nematodes from foot tissues during dissections.

### 3.2 Histopathology

**Fig. 1:**
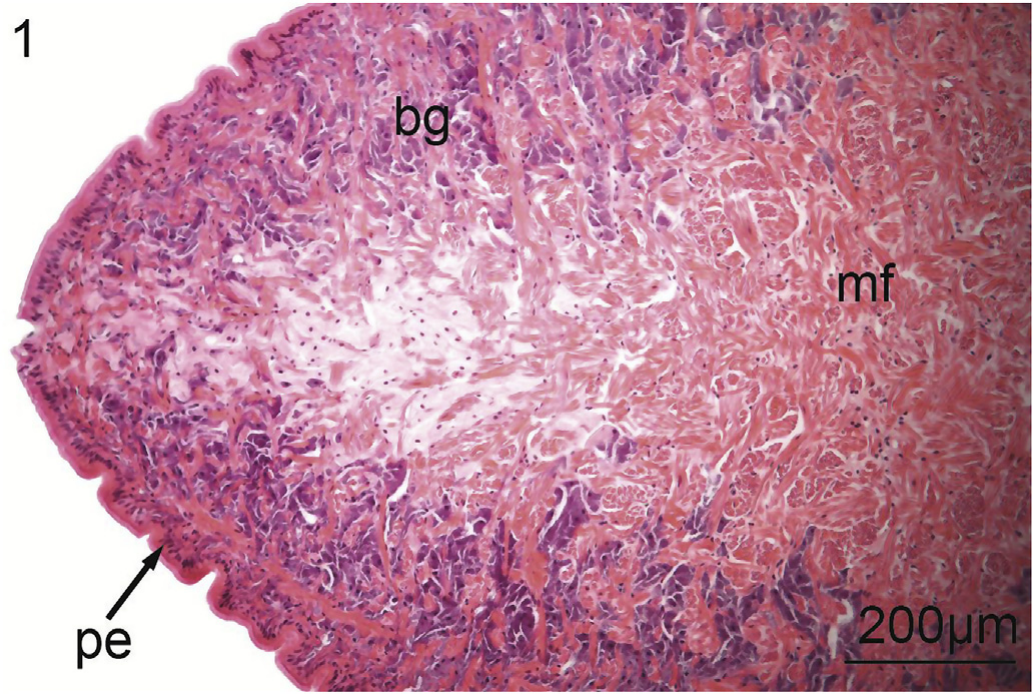
Ventral portion of an uninfected foot of *Villosa nebulosa* showing myofibers (mf), basophilic granulocytes (bg), and pedal epithelium (pe).

**Fig. 2:**
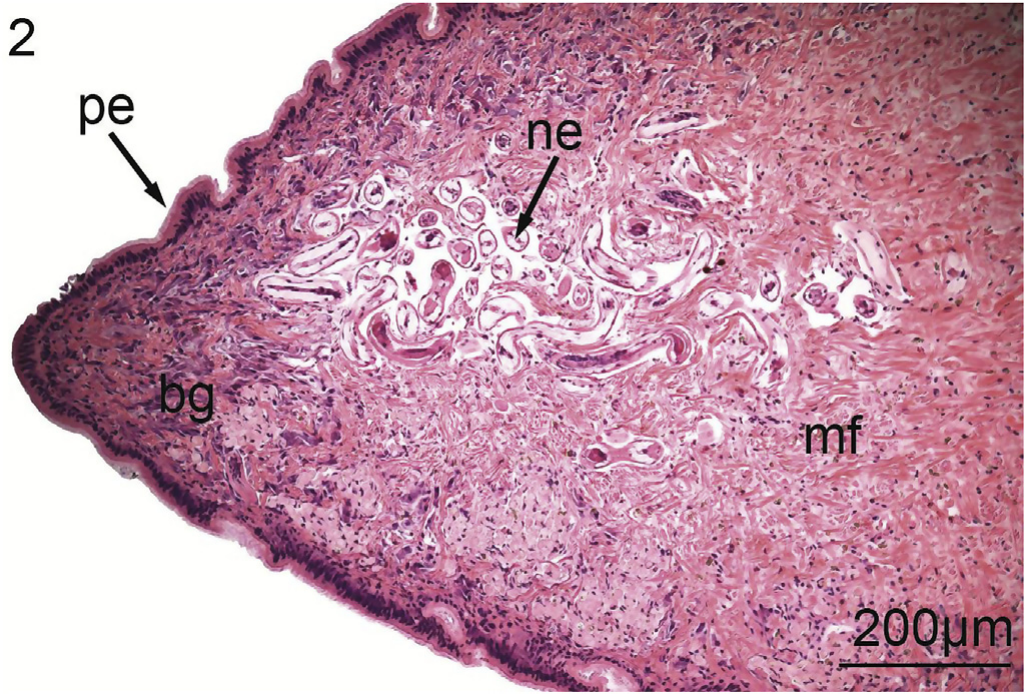
Ventral portion of infected foot of *Villosa nebulosa* showing a nematode infection (ne), myofibers (mf), basophilic granulocytes (bg), and pedal epithelium (pe).

**Fig. 3:**
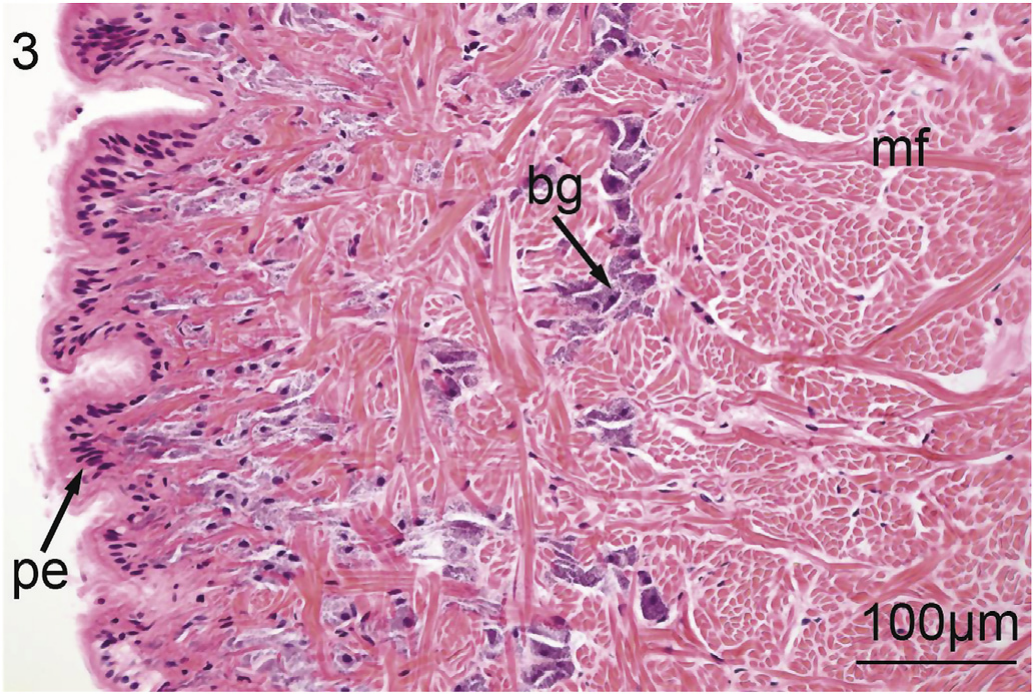
Ventro-lateral aspect of an uninfected foot of *Villosa nebulosa* showing overlapping bundles of myofibers (mf), basophilic granulocytes (bg), and ciliated pedal epithelium (pe).

**Fig. 4:**
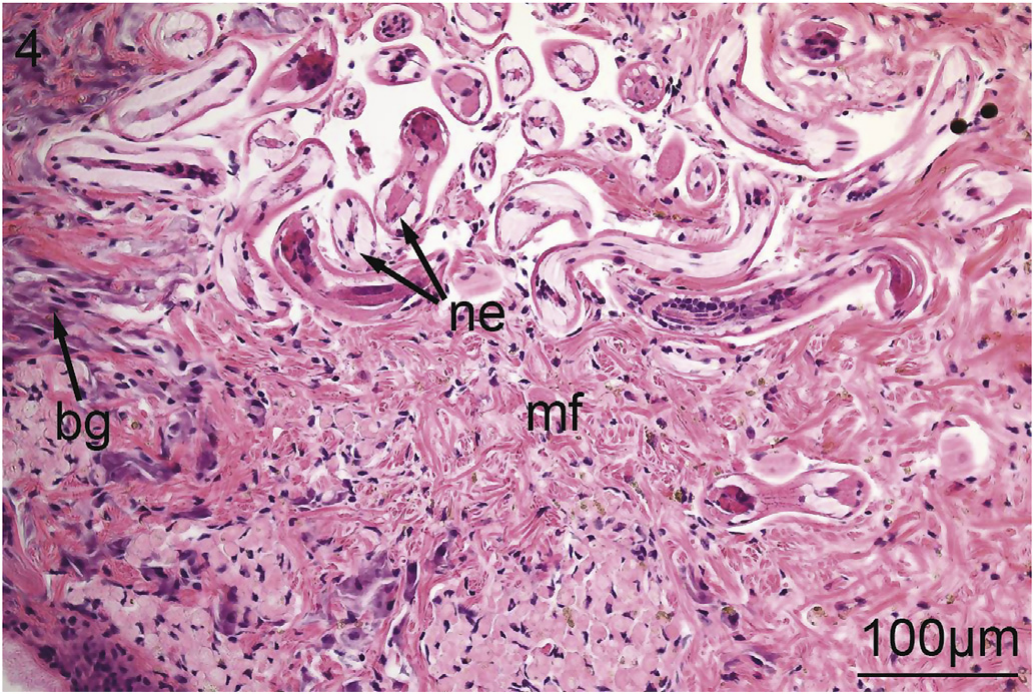
Ventro-lateral portion of an infected foot of *Villosa nebulosa* showing a nematodes (ne), myofibers (mf), basophilic granulocytes (bg).

**Fig. 5:**
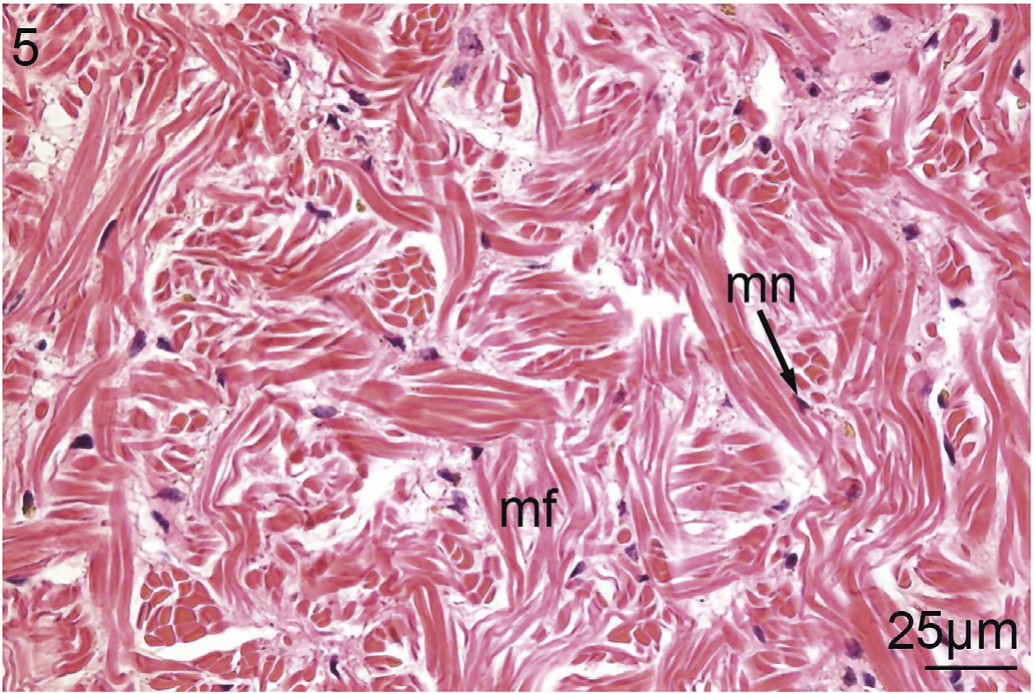
Uninfected foot of *Villosa nebulosa* emphasizing myofibrils (mf) and myocyte nuclei (mn).

**Fig. 6:**
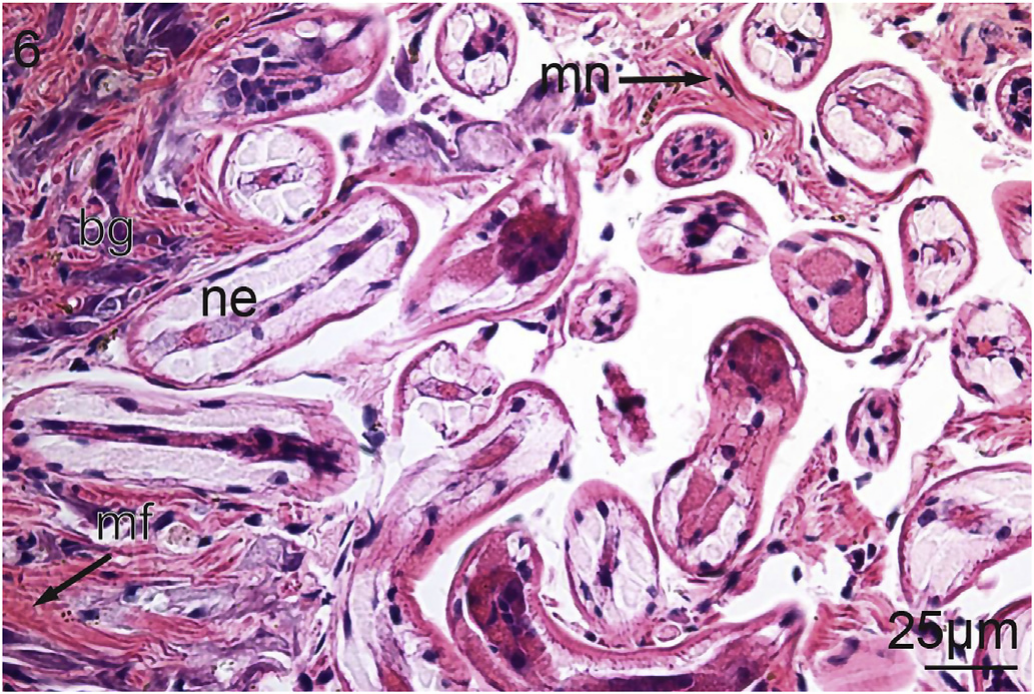
Infected foot of *Villosa nebulosa* showing nematodes (ne), myofibrils (mf), myocyte nuclei (mn), and basophilic granulocytes (bg).

The foot of *V. nebulosa* is mainly composed of bundles of somatic muscle that become branched near the ventral margin. Hematoxylin and eosin stained sections of foot revealed the presence of two distinct groups of cells having a granular cytoplasm. In the medial portion of the foot, there are pale, basophilic granulocytes while darker, violet, basophilic granulocytes are located laterally and ventrally (Fig. 1). Nematodes were principally located medially in the ventral region of the foot (Fig. 2). Pedal musculature of *V. nebulosa* is well organized with large fascicles medially and the myofibers overlap as they near the ciliated epithelium (Fig. 3). Infected tissue typically displayed an irregular, medial-ventral gap in the somatic musculature containing roundworms (Fig. 4). Nematodes were typically concentrated in this area, but were occasionally isolated in more dorsal and lateral regions of the foot. At a higher magnification, myofibrils appear to be densely packed and intricately interlaced (Fig. 5). Worms were typically arranged in different orientations. Surrounding the focus of worms, the myofibers and granulocytes were intact and histologically indistinguishable from uninfected tissue (Fig. 6). At high magnification, roundworms were closely positioned to myofibrils and the tissue locally conformed to the curvature of the worms. The medial aspect of the infected area contained a small amount of fibrous debris (Fig. 6). A cellular response to the nematodes was not observed.

**Fig. 7:**
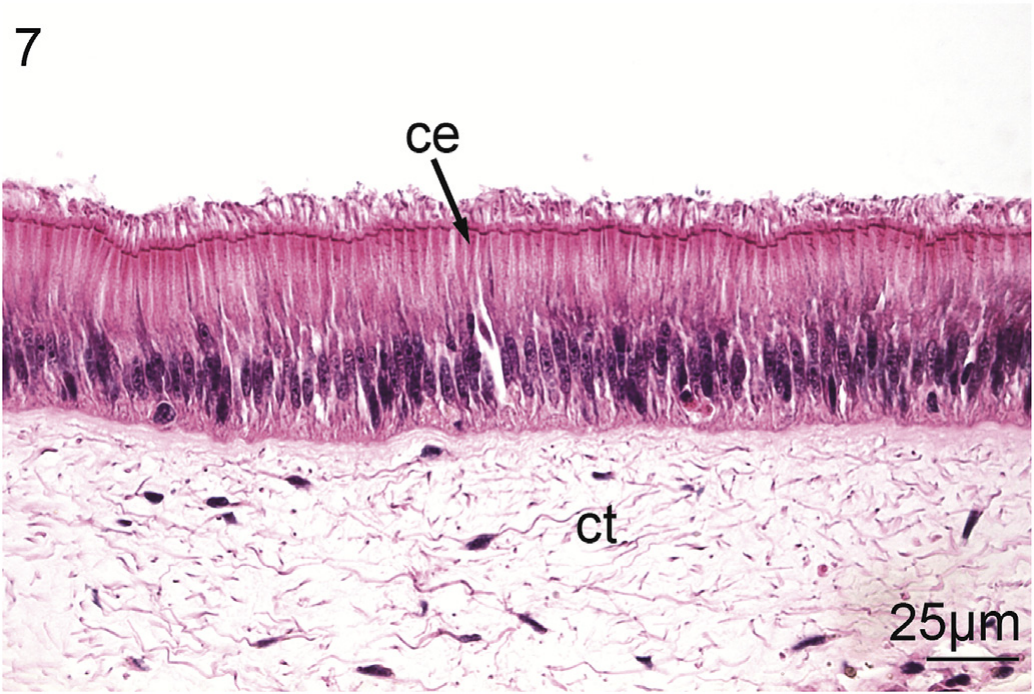
Uninfected intestine of *Villosa nebulosa* showing ciliated columnar epithelium (ce), and connective tissue (ct).

**Fig. 8:**
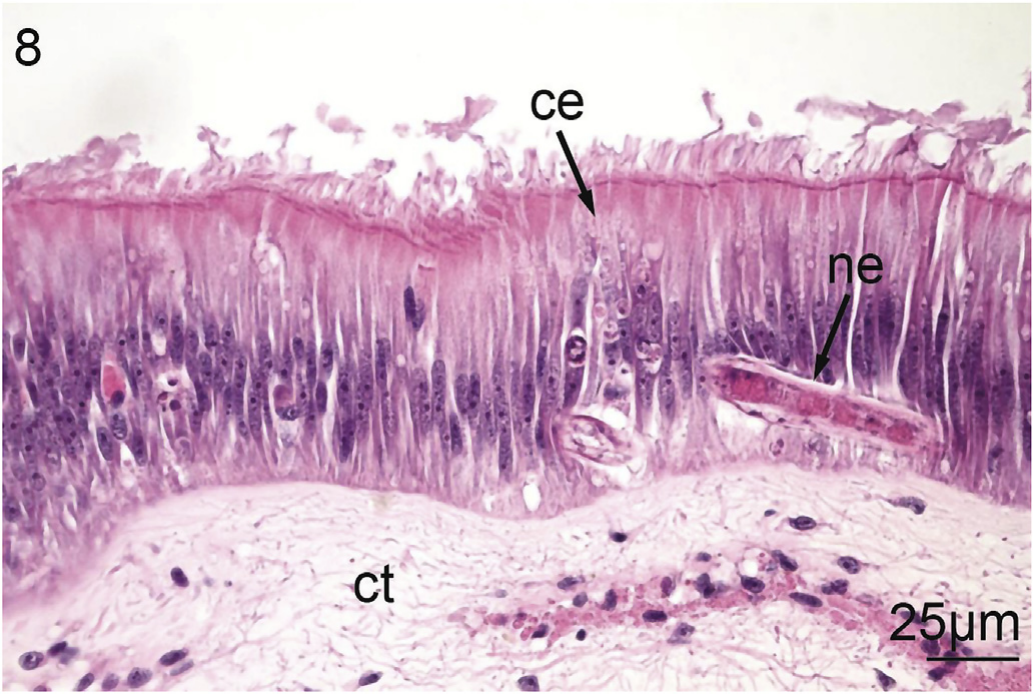
Infected intestine of *Villosa nebulosa* showing ciliated columnar epithelium (ce), connective tissue (ct), and a nematode (ne).

Intestinal nematodes were located within the epithelium of the fourth limb of the intestine, which is characterized by a major typhlosole in the dorsal aspect of the visceral mass (McElwain and Bullard, 2014). Uninfected intestinal tissue was characterized, as typically, by a simple, ciliated columnar epithelium. Some parts of this epithelium may be pleated. The columnar epithelium also contains teardrop-shaped columnar cells that contain eosinophilic granules. The epithelium is surrounded by a lamina propria and loose connective tissue resembling adipocytes (Fig. 7). Nematodes always appeared to be threaded through the columnar epithelium and infected cells were intact with little cellular changes apparent except for a small, irregular gap surrounding the worms – potentially an artifact of histological processing (Fig. 8).

The literature contains limited information about tissue damage or potential host responses associated with nematodes infecting bivalves. Pathological changes to infected tissues have mainly been reported as localized discolorations or cysts in marine bivalves (Table 1). Shipley and Hornell (1904) reported specimens of *Ascaris meleagrinae* encysted in gonad, mantle, stomach and mouth and *Echinocephalus uncinatus* encysted in the adductor of *Pinctada imbricata.* Shipley and Hornell (1904) also stated that *E. uncinatus* may occasionally become embedded in the nacre. Encysted *E*. *uncinatus* have also been reported from the adductor of *Placuna placenta* (Willey, 1907) and from unspecified tissues of *Pinna* sp. (Baylis and Oxon, 1920). In one case, an unidentified nematode appeared to have been encased within a pearl in *P. placenta* (Herdman and Hornell, 1906). Tissue discolorations associated with nematodes have been documented by Shipley and Hornell (1904), Ko et al. (1975), Lester et al. (1980), Lichtenfels et al. (1980), McLean (1983), Deardorff (1989). Pink cysts in the adductor of *Pinctada margaritifera* contained larvae of *E. uncinatus* (Shipley and Hornell, 1904). Ko et al. (1975) observed green spots around *E. sinensis* infecting the digestive diverticula, stomach, intestine, and mantle of *Magallana gigas*. Lester et al. (1980) reported a brown discoloration to the adductor of *Spisula solidissima* infected with *Sulcascaris sulcata*. *S. sulcata* was also associated with an unspecified color change to the gonad of *A. gibbus* (Lichtenfels et al., 1980). The gonad of *A. gibbus* typically becomes bright orange when they are ready to spawn (Miller et al., 1979). Yellow-brown spots occurred in the adductor of *A. ventricosus* infected with *E. pseudouncinatus* (McLean, 1983). *S. sulcata* was also associated with a yellow-brown discoloration of the adductor of *A. gibbus* (Deardorff, 1989). Brownish spots indicated the presence of encapsulated nematodes in *Tagelus plebeius* (Vázquez et al., 2006). It remains uncertain whether tissue discolorations are the result of cellular damage, immunological responses to parasites or secretions from parasites. In some cases, nematodes may be pigmented, but it is unclear if this coloration contributes to the coloration of infected tissue (Shipley and Hornell, 1904; Cummins, 1971; Perkins et al., 1975; Lichtenfels et al., 1978; Cannon, 1978). Other observations of gross pathological changes to tissue have included caseous tissue near the adductor of *A. balloti* associated with larval ascaridoid worms (Cannon, 1978). Also, Payne et al. (1980) reported a slight thickening of the infection site associated with nematodes presumed to be a *Sulcascaris* sp. in *S. solidissima*, and some worms occurred in watery cysts. In the present investigation, we did not observe any obvious gross pathological changes to the tissues of *Villosa nebulosa* infected with nematodes*.*

Parasitological investigations, in which histology was a focus, have provided little insight into the cellular changes to host tissues that occur during a roundworm infection. Burton (1963) observed small nematodes measuring 75 μm in diameter within the digestive diverticulum. Burton (1963) also reported dense concentrations of leukocytes, encapsulation responses, and gastric and intestinal ulcers, but it was unclear whether such observations were associated with nematode infections or not. Cheng (1966) experimentally infected *Crassostrea virginica* with *Angiostrongylus cantonensis*. A preponderance of hemocytes was observed within and around hemolymph vessels of infected oysters as compared to uninfected oysters. Leukocytes surrounded nematodes between 10 and 14 days post infection. Cheng (1967) reported unidentified nematodes coiled near digestive diverticula of *C. virginica*, but no other details were provided. Cheng (1975a) reported *E. crassostreai* from *Magallana gigas*. *E. crassostreai* did not cause appreciable histopathological changes to the gonoduct lining, but a 0.15 mm tunic of connective tissue fibers, hemocytes, myofibers and Leydig cells were observed around the gonoducts. Nematodes infecting the ovaries were associated with displaced, shrunken, ruptured or compressed ova. In a follow-up study, Cheng (1975b) reported brown cells in the reaction complex, but the function of these cells remains unclear. Ko et al. (1975) reported *E*. *sinensis* from *M. gigas*. Worms were located in Leydig tissue and gonoducts of male and female oysters. Intensity of tissue reactions varied from no apparent host response to a conspicuous response. Host responses included infiltration of amoebocytes around the worms and extensive fibroplasia. Infected oysters also displayed enlarged gonoduct lumen, desquamation, erosion of ciliated epithelium and metaplasia of pseudostratified columnar epithelium into cuboidal or squamous epithelium. Cannon (1978) observed an encapsulation response to larval ascaridoid worms infecting the adductor of *A. balloti*. An encapsulation response occurred in histological sections of the adductor of *A. gibbus* infected with *S. sulcata* (Deardorff, 1989). Murchelano and MacLean (1990) provided an update on the histopathological changes associated with nematodes in bivalves reported by Burton (1963), Cheng (1966), Lichtenfels et al. (1978), and Perkins et al. (1975) with higher resolution images of *S. sulcata* infecting *S. solidissima* and unidentified nematodes infecting *C. virginica*. Kim and Powell (2004) observed unidentified nematodes infecting the digestive gland, visceral mass between body wall and underlying muscle tissue, foot, muscle tissue, gill of *S. solidissima*. Nematodes were frequently associated with hemocytic infiltration (Kim and Powell, 2004). Vázquez et al. (2006) reported nematodes infecting *T. plebeius* were either free or encapsulated in tissues. The capsule consisted of a dense aggregation of hemocytes and sometimes bundles of muscle fibers appeared to comprise the outer capsule wall.

Irrespective of whether *V. nebulosa* individuals exhibited a low or high infection intensity, there did not appear to be a cellular response to nematode presence. Metazoan parasites infecting bivalves are sometimes associated with an encapsulation response characterized by hemocytes surrounding the parasite and, in some cases, fibrosis (Pauley and Becker, 1968; Cheng and Rifkin, 1970; Huehner and Etges, 1981; Feng, 1988). The lack of a host response associated with nematodes in *V. nebulosa* may be indicative of some form of immunological suppression (Sorci et al., 2013).

### 3.3 Morphological and molecular taxonomic identification of the nematode

**Fig. 9:**
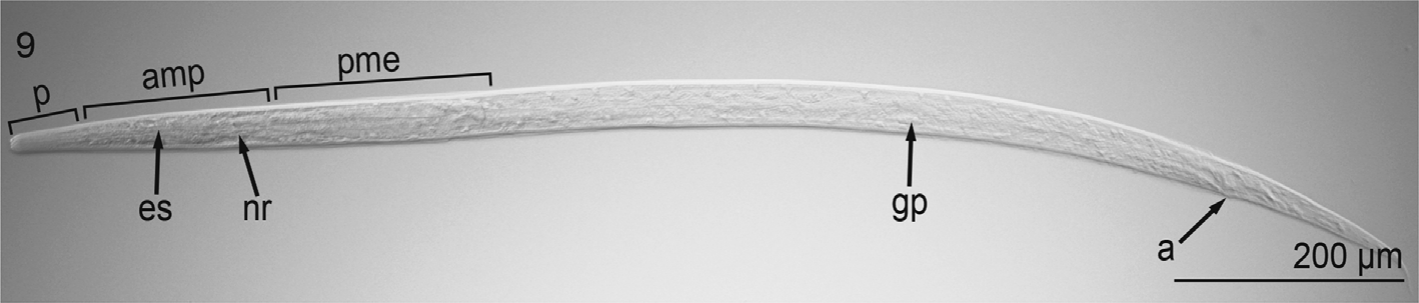
Second-stage larva of Ascaridomorpha sp. (Nematoda) infecting *Villosa nebulosa*, in lateral view. Body showing tripartite esophagus ([es], 1st part representing pharynx [p], 2nd part representing muscular anterior portion [amp], 3rd part representing muscular posterior portion [pme]), nerve ring (nr), genital primordium (gp), and anus (a).

**Fig. 10:**
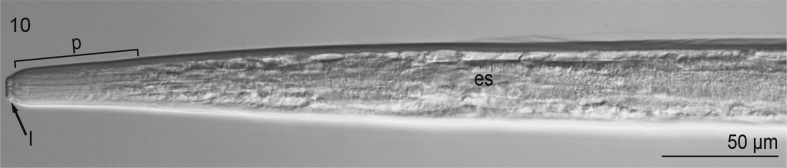
Second-stage larva of Ascaridomorpha sp. (Nematoda) infecting *Villosa nebulosa*, in lateral view. Anterior end of body showing lips (l), pharynx (p), and esophagus (es).

**Fig. 11:**
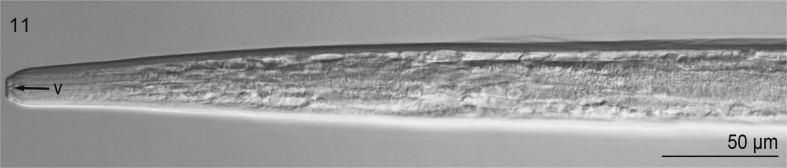
Second-stage larva of Ascaridomorpha sp. (Nematoda) infecting *Villosa nebulosa*, in lateral view. Anterior end of body showing vestibule (v).

**Fig. 12:**
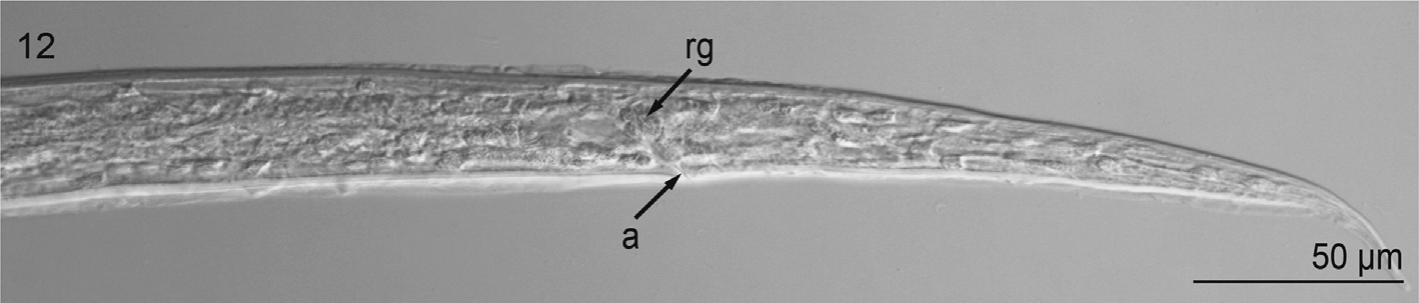
Second-stage larva of Ascaridomorpha sp. (Nematoda) infecting *Villosa nebulosa*, in lateral view. Posterior end of body showing anus (a), rectal glands (rg).

Based on 6 cleared, whole-mounted specimens (Fig. 9, Fig. 10, Fig. 11, Fig. 12) (USNM 1568349–1568350) observed and measured (mean ± s.d., n are presented below) at 100 × objective magnification using differential interference contrast optical components: Body minute, extremely slender or hair-like, 1000–1185 μm (1104 ± 72, 6) long, 33–36 μm (35 ± 1.3, 6) wide, colorless, having smooth cuticle, lacking cuticular alae (Fig. 9). Cephalic end blunt, slightly rounded, with 2 discernible labia (1 dorsal and 1 ventral); short vestibule present (Fig. 10). Excretory pore not observed. Esophagus tripartite (anterior pharynx followed by 2 muscular sections), rhabditiform but lacking bulb at base (Fig. 9, Fig. 10); 160–200 μm (179 ± 16, 6) long and 12–14 μm (13 ± 0.8, 6) wide. Nerve ring 125–135 μm (130 ± 3.5, 6) from anterior extremity. Intestine nearly indistinct, straight, narrow for entire length. Genital primordium in hindbody, approximately 400 μm from tip of tail (Fig. 9). Rectal glands subspherical, nearly indistinct (Fig. 11). Tail 160–185 μm (173 ± 10.3, 6) long, conical, slender, with pointed tip (lacking droplet shaped tip) (Figs. 9,11,12).

The minute size of and lack of discernible genitalia and demonstrable cuticular features in our specimens indicated that they were larval specimens (Moravec, 1998). The presence of a genital anlagen (primordium) comprising a single small cell strongly suggested that they represented a second stage larva, L2 because the genital anlagen develops and differentiates late in the L3 or during the L4. Further, given that they comprised unencysted, unencapsulated larvae infecting a mollusc (rather than a vertebrate), we suspect that they may comprise an L2. As such, it was not possible to diagnose them to a family; however, noteworthy is that they demonstrated morphological features consistent with larval specimens of species of both Seuratoidea and Cosmocercoidea (Anderson, 2000; i.e., Cosmocercoidea: having esophagus with three discernible sections, including an anteriorly distinct pharynx and slender tail; Seuratoidea: esophagus lacking bulb at its base). However, confident diagnosis based upon morphology alone is tenuous because the larval types for species of these superfamilies have not been morphologically diagnosed. These larval nematodes may represent an innominate taxon.

**Fig. 13:**
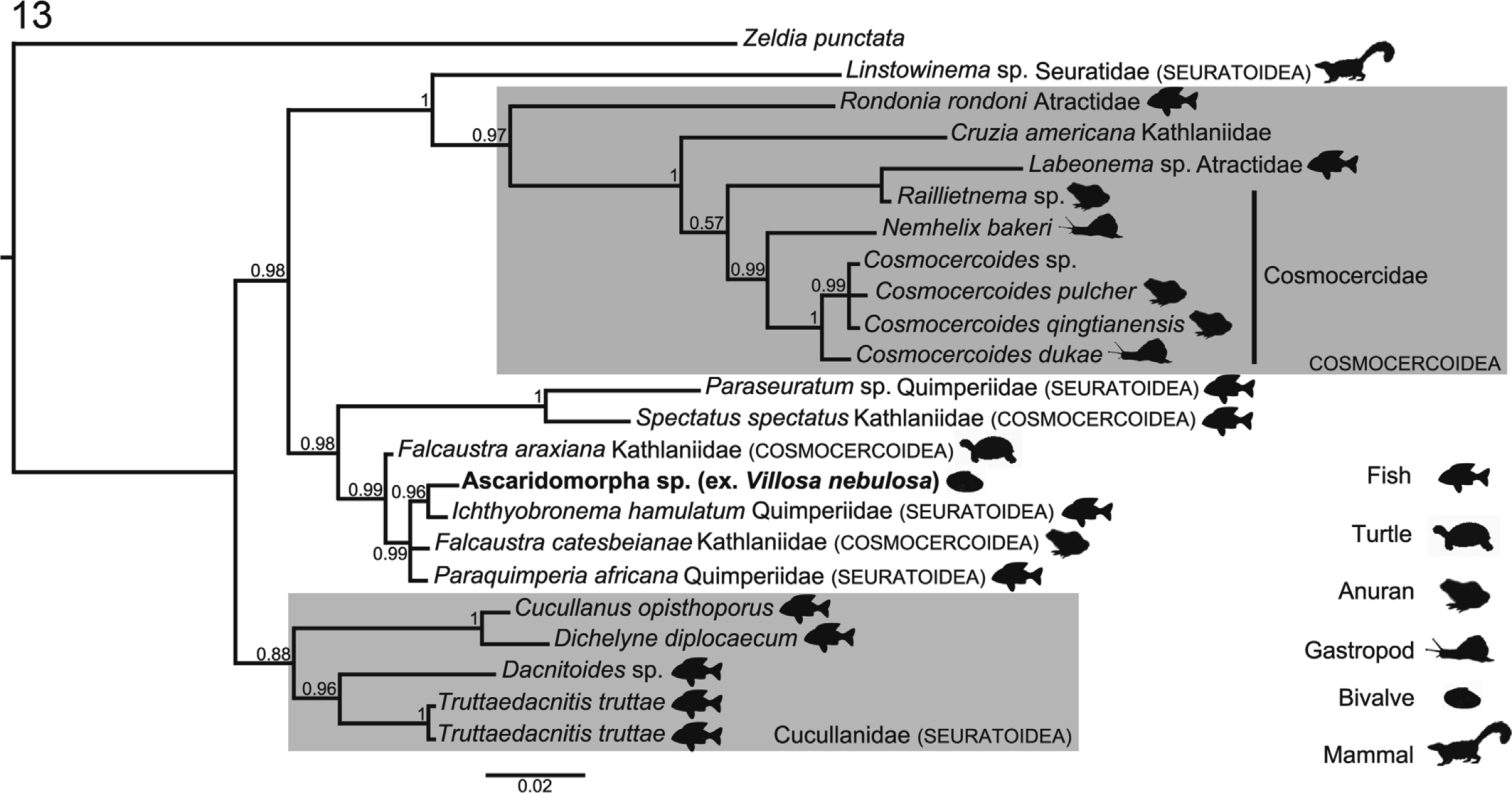
Phylogenetic interrelationships of nematodes (Cosmocercoidea, Seuratoidea) based on sequences of the 18S rDNA, generated from Bayesian inference. Nodal supports were estimated by Bayesian posterior probability (BPP) after running the Markov chain Monte Carlo (2 runs 4 chains, 4 × 10^6^ generations, sampling frequency = 4 × 10^3^, burn-in = 1 × 10^6^). Sequence obtained in the present study is in bold.

The sequence of the small subunit rDNA (18S; 835 base pairs; GenBank Accession Number: MK959030) from the nematode infecting *V. nebulosa* was 99% similar to that of 4 ascaridid nematodes accessioned in GenBank (Table 3). The phylogenetic analysis recovered them all as sharing a recent common ancestor: the nematode infecting *V. nebulosa* was sister to *Ichthyobronema hamulatum* (Seuratoidea: Quimperiidae; GenBank Accession Number KY476351 ex. burbot, *Lota lota* [Linnaeus, 1758] [Gadiformes: Lotidae] from Russia) (Sokolov and Malysheva, 2017) (Fig. 12). These nematodes were recovered within a polytomy including *Paraquimperia africana* (Seuratoidea: Quimperiidae; JF803925; ex. giant mottled eel, *Anguilla marmorata* [Quoy and Gaimard, 1824] [Anguilliformes: Anguillidae] from South Africa) (Moravec, 2007) and *Falcaustra catesbeianae* (Cosmocercoidea: Kathlaniidae; AB818380; ex. bullfrog, *Lithobates catesbeianus* [Shaw, 1802] [Anura: Ranidae] from Japan) (Hasegawa et al., 2013) (Fig. 12). All of these taxa were sister to *Falcaustra araxiana* (Kathlaniidae; KM200715; ex. European pond turtle, *Emys marmorata* [Linnaeus, 1758] [Cryptodira: Emydidae] from Iran) (Rajabloo et al., 2016), forming a well-supported clade (Fig. 13).

**Table 3:** Species whose sequences of the 18S rDNA were retrieved from GenBank and used for phylogenetic analysis.

Species	Host	Geographic origin	Accession number
*Cosmocercoides dukae* (Holl, 1928)	*Deroceras panormitanum* (Lassona and Pollonera, 1822)	USA	FJ516753
*C*. *pulcher* Wilkie, 1930	*Bufo japonicas* Temminck and Schlegel, 1838	Japan	MH178322
*C*. *qingtianensis* Chen, Zhang, Nakao and Li, 2018	*Bufo gargarizans* Cantor, 1842	China	MH032769
*Cosmocercoides* sp.	Not reported	Not reported	MK110872
*Cruzia americana* Maplestone, 1930	Not reported	Not reported	U94371
*Cucullanus opisthoporus*Pereira and Luque (2017)	*Cichla pinima* Kullander and Ferreira, 2006	Brazil	KX752096
*Dacnitoides* sp.^a^	*Scomberoides lysan* (Forsskål, 1775)	India	KJ566940
*Dichelyne diplocaecum* Chandler, 1935^b^	Not reported	Mexico	HQ241925
*Falcaustra araxiana* Massino, 1924	*Emys orbicularis* (Linnaeus, 1758)	Iran	KM200715
*Falcaustra catesbeianae* Walton, 1929	*Lithobates catesbeianus* (Shaw, 1802)	Japan	AB818380
*Ichthyobronema hamulatum* (Moulton, 1931)	*Lota lota* (Linnaeus, 1758)	Russia	KY476351
*Labeonema* sp.	*Synodontis ocellifer* Boulanger, 1900	Senegal	EF375487
*Linstowinema* sp.	*Isoodon obesulus* (Shaw, 1797)	Tasmania	JF934727
*Nemhelix bakeri* Morand and Petter, 1986	*Helix aspersa* (Müller, 1774)	UK	DQ118537
*Paraquimperia africana* Moravec, Boomner and Taraschewski, 2000	*Anguilla marmorata* Quoy and Gaimard, 1824	South Africa	JF803925
*Paraseuratum* sp.	*Hoplias microlepis* (Günther, 1864)	Panama	KP275686
*Raillietnema* sp.	*Ceratobatrachus guentheri* Boulenger, 1884	Canada	DQ503461
*Rondonia rondoni* Travassos, 1920	*Pterodoras granulosus* (Valenciennes, 1821)	Peru	DQ442679
*Spectatus spectatus* Travassos, 1923	*Piaractus mesopotamicus* (Holmberg, 1887)	Brazil	KR139827
*Truttaedacnitis truttae* (Fabricius, 1794)^c^	*Oncorhynchus mykiss* (Walbaum, 1792)	USA	KP275682/EF180063
*Zeldia punctata* (Thorne, 1925)	Free living	USA	ZPU61760

a Validity of the genus is questionable.

b Named as *Dichelyne mexicanus* in GenBank; valid name is *D. diplocaecum*.

c Some authors consider *Cucullanus truttae* as valid name.

As an aside, Cosmocercoidea and Seuratoidea (and their respective families) were paraphyletic or polyphyletic in the recovered tree, and Cucullanidae was recovered as an early branching lineage (monophyletic). These results corroborate earlier findings regarding the phylogenetic interrelationships of these taxa (Pereira et al., 2015; Choudhury and Nadler, 2016; Sokolov and Malysheva, 2017). The superfamilies Seuratoidea (Cucullanidae, Quimperiidae, Seuratidae, et al.) and Cosmocercoidea (Atractidae, Cosmocercidae, Kathlaniidae) are little studied regarding their life cycles and phylogenetic interrelationships (both groups exhibited paraphyly or polyphyly herein). Quimperiidae (Seuratoidea) and Kathlaniidae (Cosmocercoidea) are especially poorly studied, and no quimperiid or kathlaniid larva has been morphologically characterized to date.

Regarding the taxonomic identification and phylogenetic placement of the nematode larvae herein, they claded within a well-supported assemblage of quimperiid and kathlaniid species (all in the Ascaridomorpha) and had morphological features of both groups. As such, combined with the available morphological and life history evidence, we conservatively identified our nematode larvae as a species within the nematode infraorder Ascaridomorpha (i.e., Ascaridomorpha sp.).

The biodiversity of nematodes associated with bivalve molluscs is poorly understood. Most records are from commercially important marine species. Many records of nematodes associated with freshwater bivalves are from *Dreissena polymorpha*, an invasive species that has become established in many water bodies throughout Europe and North America (Karatayev et al., 2000, 2003; Quinn et al., 2014). The lack of a specific identity for nematodes associated from bivalves reported in the literature may be an indication that nematodes are difficult to identify as larvae because species-specific morphological characters are underdeveloped. Additionally, it may not be feasible to identify nematodes from histological sections of intermediate hosts potentially because their small size makes them difficult discern during routine microtomy and because worms may be coiled or arranged in a sinuous manner making it laborious to characterize their entire anatomy (e.g., Burton, 1963; Deardorff, 1989; Murchelano and MacLean, 1990; Vázquez et al., 2006).

Our observations represent the first description of a nematode species that invades the tissues of a Unionidae species. Clark and Wilson (1912), Wilson and Clark (1912), and Coker et al. (1921) reported “*Ascaris* sp.” and “*Ascaris*-like” nematodes infecting North American unionids and or margaritiferids; however, these authors did not morphologically diagnose the nematodes and, to the best of our knowledge, no specimen was deposited in a curated museum. Although we attempted to morphologically diagnose our nematode specimens infecting *V. nebulosa* to family, the morphological features of these larval specimens were inadequate to do so. Moreover, the molecular phylogenetic results were equivocal, to some extent, based on the small fragment (835 bp) and the self-evident systematic revision needed among the Quimperiidae, Kathlaniidae and other families of Cosmocercoidea and Seuratoidea, or whether these superfamilies are valid (all of which were recovered as paraphyletic or polyphyletic in the 18S phylogeny). While the prevalence among the sampled mussels was high, we do not know if these nematodes are locally abundant in the South Fork of Terrapin Creek or how common these nematodes are throughout the range of *V. nebulosa*.

## 4 Conclusions

Since this study was based on field collections, there are several gaps in our understanding of the host-parasite relationship. The route of infection is presently indeterminate; we are uncertain if eggs or larvae are ingested by mussels or if larvae penetrate the integument from the surrounding sediment and migrate to other tissues. Secondly, we do not know whether *V. nebulosa* represents a paratenic or intermediate host. A wide range of predators, some of which are potential definitive hosts, may feed on freshwater mussels. Therefore, mussels may be indicators of ecosystem health (Haag, 2012). Additionally, it is unclear to what extent nematodes may impair the function of host tissues. For example, in mussels in which we observed a large number of nematodes, we are uncertain if pedal extension and or retraction was impaired. Unionids use their foot to burrow into specific sediments and the impairment of pedal extension and retraction could make them vulnerable to becoming dislodged.
